# Cost-effectiveness of vaccination of immunocompetent older adults against herpes zoster in the Netherlands: a comparison between the adjuvanted subunit and live-attenuated vaccines

**DOI:** 10.1186/s12916-018-1213-5

**Published:** 2018-12-06

**Authors:** Pieter T. de Boer, Alies van Lier, Hester de Melker, Albert J. M. van Wijck, Jan C. Wilschut, Albert Jan van Hoek, Maarten J. Postma

**Affiliations:** 1 Centre for Infectious Disease Control, National Institute for Public Health and the Environment, Antonie Van Leeuwenhoeklaan 9, 3721 MA Bilthoven, The Netherlands; 2 Unit of PharmacoTherapy, -Epidemiology & -Economics (PTE2), University of Groningen, Groningen Research Institute of Pharmacy, Groningen, The Netherlands; 3 Pain Clinic, University Medical Centre Utrecht, Utrecht, The Netherlands; 4 Department of Medical Microbiology, University Medical Center Groningen, University of Groningen, Groningen, The Netherlands; 5 Department of Infectious Disease Epidemiology, Faculty of Epidemiology and Population Health, London School of Hygiene & Tropical Medicine, London, UK; 6 Department of Health Sciences, University of Groningen, University Medical Center Groningen, Groningen, The Netherlands; 7 Department of Economics, Econometrics & Finance, University of Groningen, Faculty of Economics & Business, Groningen, The Netherlands

**Keywords:** Herpes zoster, Cost-effectiveness, Vaccination, Post-herpetic neuralgia, Subunit vaccine, Live-attenuated vaccine

## Abstract

**Background:**

The newly registered adjuvanted herpes zoster subunit vaccine (HZ/su) has a higher efficacy than the available live-attenuated vaccine (ZVL). National decision-makers soon need to decide whether to introduce HZ/su or to prefer HZ/su above ZVL.

**Methods:**

Using a Markov model with a decision tree, we conducted a cost-effectiveness analysis of vaccination with HZ/su (two doses within 2 months) or zoster vaccine live (ZVL) (single dose, or single dose with a booster after 10 years) for cohorts of 50-, 60-, 70- or 80-year-olds in the Netherlands. The model was parameterized using vaccine efficacy data from randomized clinical trials and up-to-date incidence, costs and health-related quality of life data from national datasets. We used a time horizon of 15 years, and the analysis was conducted from the societal perspective.

**Results:**

At a coverage of 50%, vaccination with two doses of HZ/su was estimated to prevent 4335 to 10,896 HZ cases, depending on the cohort age. In comparison, this reduction was estimated at 400–4877 for ZVL and 427–6466 for ZVL with a booster. The maximum vaccine cost per series of HZ/su to remain cost-effective to a willingness-to-pay threshold of €20,000 per quality-adjusted life year (QALY) gained ranged from €109.09 for 70-year-olds to €63.68 for 50-year-olds. The cost-effectiveness of ZVL changed considerably by age, with corresponding maximum vaccine cost per dose ranging from €51.37 for 60-year-olds to €0.73 for 80-year-olds. Adding a ZVL booster after 10 years would require a substantial reduction of the maximum cost per dose to remain cost-effective as compared to ZVL single dose. Sensitivity analyses on the vaccine cost demonstrated that there were scenarios in which vaccination with either HZ/su (two doses), ZVL single dose or ZVL + booster could be the most cost-effective strategy.

**Conclusions:**

A strategy with two doses of HZ/su was superior in reducing the burden of HZ as compared to a single dose or single dose + booster of ZVL. Both vaccines could potentially be cost-effective to a conventional Dutch willingness-to-pay threshold for preventive interventions. However, whether HZ/su or ZVL would be the most cost-effective alternative depends largely on the vaccine cost.

**Electronic supplementary material:**

The online version of this article (10.1186/s12916-018-1213-5) contains supplementary material, which is available to authorized users.

## Introduction

Herpes zoster (HZ) is a painful, itchy, vesicular rash with a characteristic dermatomal distribution. It is caused by the reactivation of latent varicella-zoster virus (VZV), previously introduced during primary infection (varicella) [1]. In the Netherlands, where varicella vaccination has not been implemented, over 95% of the population is infected with VZV before the age of 6 years [2]. The average incidence of HZ in the Netherlands was 520 per 100,000 person-years in the period 2012–2015, and older age and a compromised immune system are the most important risk factors [1, 3, 4]. However, given the ageing population, it is expected that the incidence of HZ will increase in the near future [5]. Persistent pain, labelled post-herpetic neuralgia (PHN), is the most common complication of HZ occurring in 3–19% of the patients [1, 6, 7]. PHN might persist for years and is associated with significant interference with daily life activities [8, 9].

Besides the prevention by vaccination, therapeutic options for HZ and PHN are scarce. Therefore, several countries, including the United Kingdom (UK) and United States (US), have introduced HZ vaccination for older adults using the existing live-attenuated vaccine (zoster vaccine live [ZVL] or Zostavax®), which has been registered in 2006 and was the only available vaccine at the time of introduction of vaccination in the UK/US. ZVL contains the live-attenuated OKA VZV strain and is registered for immunocompetent adults aged ≥ 50 years [10]. A large randomized clinical trial among older adults aged ≥ 60 years showed that a single dose of ZVL was 51% (95% confidence interval [95% CI] 44–58%) efficacious against HZ and 67% (95% CI 48–79%) against PHN [11]. However, efficacy against HZ was limited in the eldest age groups (38% [95% CI 25–48%] among ≥ 70-year-olds), and a long-term follow-up study demonstrated that protection had completely waned within 10 years [12]. Therefore, several countries, including the Netherlands, decided against the use of ZVL in a public programme [13]. Nowadays, several post-licensure studies have confirmed the safety and effectiveness of ZVL. However, the decline of effectiveness with increasing age was less evident in post-licensure studies as compared to the clinical trial [14–17].

A new adjuvanted HZ subunit vaccine (HZ/su or Shingrix®) might potentially overcome the shortcomings of ZVL. In Europe, HZ/su has been registered for all adults aged ≥ 50 years in 2018 and is a recombinant vaccine containing the VZV glycoprotein E adjuvanted with the AS01_B_ system [18]. The vaccine has been developed to be given in a two-dose schedule given 2–6 months apart. Two large randomized clinical trials have demonstrated that the protective efficacy of two doses HZ/su against HZ incidence was 97% (95% CI 94–99%) among immunocompetent ≥ 50-year-olds and 91% (95% CI 87–95%) among ≥ 70-year-olds [19, 20]. The duration of protection is currently unknown, but the trial confirmed a relative stable efficacy over 4 years of follow-up [20].

Due to the registration of HZ/su, governments have the option to reassess their vaccination policy for HZ in the near future, but cost-effectiveness analyses for HZ/su are yet scarce. Previous cost-effectiveness studies for the US, Hong Kong and Germany exist [21–24]; however, results from other countries are not directly transferable to the Dutch context due to potential differences in epidemiological conditions, existing vaccination policies against HZ and varicella, healthcare resource use and relative prices. Moreover, no comparison of HZ/su with ZVL booster strategies has been performed, and a lack of studies incorporating real-world effectiveness data for ZVL is noticed. Therefore, we conducted a cost-effectiveness analysis of HZ vaccination for the Dutch setting. First, we compared the impact of HZ/su and ZVL in immunocompetent older adults using efficacy data from the original vaccine trials, and we included a booster alternative for ZVL as well. Next, we analysed the threshold cost-effectiveness of HZ vaccination by seeking the maximum vaccine cost allowed to remain below €20,000 per quality-adjusted life year (QALY) gained, which is the conventional cost-effectiveness threshold for vaccination in the Netherlands [25]. As the burden of HZ and vaccine efficacy against HZ depends on age, we studied HZ vaccination for different vaccination ages and explicitly addressed the use of ZVL post-licensure real-world effectiveness data in a sensitivity analysis.

## Methods

### Overview

A static Markov model connected with a decision tree (Additional file 1: Figure S1) was developed in Microsoft Excel 2010 (Microsoft Corporation, Redmond, WA, USA) to quantify the costs and health effects (in QALYs) of vaccination of Dutch older adults against HZ from a societal perspective. We used a static model, as HZ is a reactivation of an existing VZV infection, and no impact on transmission dynamics like herd effects is expected. Studied alternatives were (i) no vaccination (current situation), (ii) vaccination with HZ/su (two doses within 2 months) and (iii) ZVL (single dose) and (iv) ZVL (single dose with a booster after 10 years). We used a 10-year period for the booster, as an immunogenicity study showed that a booster after this period is safe and provokes a similar immunological response as a first dose [26]. In the Markov model, cohorts were followed in annual cycles with an age maximum of 110 years. In each cycle, participants had the probability to stay alive or die due to another cause. Individuals who remained alive entered the decision tree, having the possibility to develop HZ, which subsequently could lead to hospitalization and/or death. We studied cohorts of 50, 60, 70 and 80 years of age. Due to the high uncertainty around the long-term efficacy of HZ/su, we limited the time horizon to 15 years in the base case analysis (for instance from 70 to 85 years of age). We chose this conservative time horizon to do justice to the low level of decline of HZ/su efficacy within the trial period but also to avoid overvaluation of the impact of vaccination among this older cohort by using a lifetime time horizon. According to the Dutch cost-effectiveness guidelines, future costs and QALYs were annually discounted by 4% and 1.5%, respectively [27].

### Epidemiology

Epidemiological inputs are shown in Table 1. Actual cohort sizes per January 1, 2017, and background mortality rates were obtained from Statistics Netherlands [28, 29]. The average incidence of HZ by age over the period 2012–2015 came from the NIVEL primary care database, a GP surveillance system covering approximately 0.7% of the Dutch population [3, 30]. We adjusted these rates for possible misdiagnoses by setting the proportion of false-positive diagnoses at 10.0% (95% CI 7.9–12.4%) [31]. Average incidence rates of HZ hospitalizations and 1-day hospital admissions by age over the period 2012–2014 were retrieved from the Dutch Hospital Data database, covering 80% of the population in 2012–2013 and 90% of the population in 2014 [32, 33]. Only admissions with a main diagnosis of HZ (International Statistical Classification of Diseases and Related Health Problems [ICD] code B02) were used. The incidence of HZ-related mortality by age was based on the number of HZ death registries (ICD-code B02) over the period 2009–2015, obtained from Dutch national death registries that contain the cause of death of 98.5% of the deaths [34]. We adjusted the mortality rates according to a validation study on HZ coding of death registries in the US, indicating that HZ was not the true underlying cause of death in 47.5% (95% CI 32.0–63.0%) of the deaths assigned to HZ [35].

**Table 1:** Model inputs of the analysis. In the probabilistic sensitivity analysis, the parameter inputs were simultaneously varied within the lower and upper estimates according to the distribution shown. In the deterministic sensitivity analysis, the parameter inputs were varied one-by-one between lower and upper inputs, while the “scenario” column shows inputs based on other plausible assumptions or sources

Variable	Base case	Lower	Upper	Distribution	Scenario	Reference (base case/scenario)
Demography						
Cohort size						Statistics Netherlands [29]
50 years	253,491					
60 years	222,845					
70 years	217,058					
80 years	93,547					
Background mortality	Age-specific					Statistics Netherlands [28]
HZ epidemiology						
HZ incidence per 100,000 person-years						NIVEL [30]/incidence adjusted for immunocompetent population using Schroder [4]
50–59 years	591	575	607	Beta	461	
60–69 years	857	835	878	Beta	669	
70–79 years	1190	1157	1222	Beta	929	
≥ 80 years	1481	1435	1527	Beta	1156	
False positive HZ diagnoses (%)	10.0	7.9	12.4	Beta	0	Van Hoek [31]/assuming no false positives
HZ hospitalization incidence per 100,000 person-years						Dutch Hospital Data [32]/incidence adjusted for the immunocompetent population using Hobbelen [49]
50–59 years	2.5	2.1	2.9	Beta	2.2	
60–69 years	4.9	4.3	5.5	Beta	4.3	
70–79 years	9.5	8.4	10.6	Beta	8.3	
≥ 80 years	18.4	16.4	20.4	Beta	16.1	
HZ 1-day hospital admission incidence per 100,000 person-years						Dutch Hospital Data [32]/incidence adjusted for the immunocompetent population using Hobbelen [49]
50–59 years	3.6	3.1	4.0	Beta	3.1	
60–69 years	9.6	8.8	10.5	Beta	8.4	
70–79 years	21.8	20.1	23.4	Beta	19.1	
≥ 80 years	28.2	25.8	30.7	Beta	24.8	
HZ mortality incidence per million person-years						Statistics Netherlands [34]/incidence adjusted for the immunocompetent population using Hobbelen [49]
50–59 years	0.1	–	0.3	Beta	0.1	
60–69 years	0.4	0.05	0.7	Beta	0.3	
70–79 years	1.8	0.9	2.8	Beta	1.6	
80–89 years	16.5	12.6	20.5	Beta	14.3	
≥ 90 years	108.9	84.6	133.2	Beta	94.5	
Misclassification HZ as underlying cause of death (%)	47.5	32.0	63.0	Beta	0	Mahamud [35]/assuming no misclassification
QALY loss						
QALY loss per HZ episode						Van Wijck [6]/utilities Van Hoek/QALY loss per HZ episode Van Hoek [31]
50–59 years	0.040	0.025	0.063	^a^	0.034/0.067	
≥ 60 years	0.057	0.039	0.093	^a^	0.053/0.200	
QALY loss per HZ death	Age-specific					Statistics Netherlands [28], Szende [36]
QALY loss grade 3 adverse event per dose						Excluded/QALY loss for grade 3 adverse events (see Additional file 1)
HZ/su	0				0.000329	
ZVL	0				0.000022	
Costs (€, 2017)						
Health care costs						
GP visit, medication, specialist visit						Based on multiple sources (see Additional file 1)
50–59 years	158	130	186	^a^		
≥ 60 years	198	163	233	^a^		
Hospital admission						
50–59 years	2856	2490	3222	^a^		
60–69 years	3632	3166	4097	^a^		
70–79 years	3671	3325	4016	^a^		
≥ 80 years	4504	4093	4915	^a^		
One-day hospital admission	282					Hakkaart-van Roijen [38]
Healthcare costs in gained life years per averted HZ death	Age-specific				0	Statistics Netherlands [28], Van Baal [40]/excluding costs in gained life years
Vaccine administration	11.36					SNPG [41]
Patient costs						
OTC medication per HZ episode						Based on multiple sources (see Additional file 1)
50–59 years	10.42	8.85	12.00	^a^		
≥ 60 years	12.65	10.75	14.56	^a^		
Travel costs GP visit, medication, specialist care per HZ episode						Based on multiple sources (see Additional file 1)
50–59 years	3.33	2.61	4.06	^a^		
≥ 60 years	4.07	3.19	4.95	^a^		
Travel costs hospital per HZ hospital visit/hospitalization	5.79					
Travel costs per vaccination	0.43					
Productivity losses						
HZ episode						Based on multiple sources (see Additional file 1)
50–59 years	398	230	744	^a^		
60–69 years	136	78	262	^a^		
≥ 70 years	0					
HZ death						Friction period of 84 working days [27]
50–59 years	14,937					
60–69 years	5074					
≥ 70 years	–					
Vaccine characteristics						
Vaccine uptake (%)	50					Assumption based on Eilers [42]
Adherence to the second dose of HZ/su (%)	100				90, 70, 50	Assumption
Efficacy HZ/su over time (linear function)						Function fitted using data from Cunningham [20], Lal [19] and Curran [47]
Intercept						
50–69 years	0.981	0.904	1.057^c^	Normal		
≥ 70 years	0.992	0.956	1.028^c^	Normal		
Slope^b^	− 0.041	0.018	0.065	Beta		
Efficacy ZVL over time (one-minus-exponential function)^d^					Additional efficacy against PHN /post-licensure effectiveness against HZ	Function fitted using data from Oxman [11], Schmader [46] and Morrison [12]/see Additional file 1
Intercept	− 0.893	− 1.04	− 0.75	Normal		
Slope	0.0807	0.058	0.104	Beta		
Risk ratio of efficacy by age						Estimated using Rohan [45] and Schmader [44]/see Additional file 1
50–59 years	1.282					
60–64 years	1.274					
65–69 years	1.219					
70–74 years	0.852					
75–79 years	0.711					
80–84 years	0.391					
≥ 85 years	0.152					

*HZ* herpes zoster, *HZ/su* HZ subunit vaccine, *PHN* post-herpetic neuralgia, *QALY* quality-adjusted life year, *SNPG* Stichting Nationaal Programma Grieppreventie, *ZVL* zoster vaccine live

^a^Aggregated costs from multiple cost items which were varied individually in the probabilistic sensitivity analysis (see Additional file 1)

^b^The slope of 0.009 was only used for 50- to 69-year-olds over the first 4 years covered by the trial. After 4 years, the slope of ≥ 70-year-olds was used

^c^The efficacy was rounded to 1 during the period that the efficacy function was above 1

^d^The efficacy of ZVL over time (in years) was modelled using a one-minus-exponential function 1 − exp(*β*_1_ + *β*_2_ × years), in which *β*_1_ is the intercept and *β*_2_ the slope. Risk ratios by age were used to modify the intercept. For instance, the efficacy of 60–64 years at time point zero was 1 − exp(1.274 × − 0.893 + 0 × 0.0807) = 67.9%. In our model, we used the VE of the age group 60–64 for vaccination of 60-year-olds, 70–74 for 70-year-olds, 80–84 for 80-year-olds and ≥ 85 years for the booster for 90-year-olds

### QALY loss

The QALY loss per HZ case was calculated by multiplying the time spent in a certain health state by the reduction of health-related quality of life (HR-QoL), i.e. disutility, associated with that health state and is shown in Table 1. For this calculation, we distinguished four health states of HZ, i.e. no pain, mild pain, moderate pain and severe pain. The proportion of patients in each health state over time was estimated using a Dutch prospective cohort study that followed HZ patients aged ≥ 50 years over a maximum period of 12 months [6]. Disutilities by health state over time were obtained from the same study and were based on the validated three-level version of the Euroqol-5 dimensions (EQ-5D-3L) instrument. As severity and duration of pain showed to increase by age, separate analyses were done for the age groups 50–59 years and ≥ 60 years. Patients with moderate or severe pain after 3 months were defined as PHN patients. We found that the risk of PHN was estimated at 2.2% for 50- to 59-year-olds and 7.3% for ≥ 60-year-olds. More details can be found in Additional file 1. Life years (LY) lost due to HZ-related premature mortality were estimated using life tables and were converted to QALYs lost using age-specific EQ-5D-3 L population norms from the Netherlands [36]. We ignored QALY losses due to vaccine-related adverse events in our base case analysis, as both HZ/su and ZVL only caused short-term symptoms, and the impact on HR-QoL was not investigated [11, 19].

### Costs

HZ-related costs per case are shown in Table 1. All costs were adjusted to the 2017 price year using the Dutch national consumer price index [37]. Costs per HZ case were estimated using the same prospective cohort study that was used to estimate HZ-related QALY losses [6]. In this study, the number of GP visits, specialist referrals, medication use and productivity loss was asked at different time points and were then converted to costs per case using standardized cost per item. Costs per hospital admission were based on the length of stay of admissions having HZ as main a diagnosis (ICD-code B02) over the period 2012–2014, and cost of a 1-day visit was obtained from the literature [32, 38]. According to the most recent Dutch guideline on cost-effectiveness research, unrelated healthcare costs in gained life years (i.e. indirect medical costs) should be included in the base case analysis [39]. These costs were estimated using the life expectancy at the age of death multiplied with yearly age-specific healthcare costs obtained from a specifically developed costing tool [40]. More details on the estimated costs per HZ episode are shown in Additional file 1. For vaccine administration costs, we used the current Dutch influenza tariff of €11.36 per dose, assuming that HZ vaccination would be provided in a GP-based programme [41]. This tariff covers, next to vaccine administration costs, costs due to patient selection and invitation, record keeping, vaccine storage and waste destruction.

### Vaccine characteristics

Vaccine-related inputs are shown in Table 1. Given that ZVL is contraindicated to immunocompromised individuals, we restricted vaccination in our analysis to the immunocompetent part of the population. A recent questionnaire among a random sample of Dutch older adults aged ≥ 50 years indicated that the acceptance rate of vaccination against HZ was 58% [42]. Considering that the proportion of the Dutch population that is immunocompromised ranges from 1.9% in 50- to 64-year-olds to 11.5% in ≥ 85-year-olds [43], we conservatively set the vaccination coverage at 50% of the total cohort. However, as we used a static model, the coverage has no impact on the cost-effectiveness outcomes of vaccination and was only used to estimate the absolute impact of vaccination such as the number of HZ cases prevented. We assumed that all HZ/su recipients received two doses of the vaccine within 2 months and that the coverage of the ZVL booster after 10 years was 50% of the cohort that was still alive at that age.

Vaccine efficacy of HZ/su and ZVL against HZ over time was modelled using data from published clinical trials (see also Additional file 1: Figure S7) [11, 19, 20, 44–46]. For both vaccines, a function of vaccine efficacy over the years was fitted to the annual efficacy data using the standard error as a weighting factor. We fitted multiple types of functions and used the functions that provided the best fit. For ≥ 70-year-olds, a linear function for two doses of HZ/su was fitted to the 4 years of trial data, resulting in a waning of 4.1% per year. We assumed that this waning rate would continue after 4 years and varied this assumption extensively in the sensitivity analysis. For 50- to 69-year-olds, a recent study estimated the waning rate at 0.9% per year using unpublished annual vaccine efficacy estimates [47]. However, no confidence intervals of these estimates were reported, and the fit of a linear function through the annual vaccine efficacy estimates was poor. Therefore, we conservatively assumed that this waning rate was only valid for the first 4 years covered by the trial and would be equal to the ≥ 70 years age group after 4 years. For ZVL, a one-minus-exponential function was fitted using follow-up data of ≥ 60-year-olds up to 11 years after vaccination [12]. To include the effect of age, we adjusted the vaccine efficacy at take using age-specific risk ratios of the clinical trial. For the ZVL booster, we assumed that the efficacy would be equal to the efficacy that an initial dose would have had at that age [26]. More details on the model fits are provided in Additional file 1.

### Effectiveness and cost-effectiveness

The effectiveness of the different HZ vaccination strategies was expressed as the number needed to vaccinate (NNV) to prevent a HZ case. The cost-effectiveness was estimated by finding the threshold vaccine cost that equals an incremental cost-effectiveness ratio (ICER) of €20,000 per QALY gained [25]. However, as this cost-effectiveness threshold does not have an official status in the Netherlands, we also show results for a threshold of €50,000 per QALY gained. The latter threshold has been used earlier for pneumococcal vaccination and is also considered by the Dutch National Health Care Institute for therapeutic interventions of diseases with a moderate disease burden that could well be comparable to PHN [25, 48].

### Sensitivity analysis

We performed a probabilistic sensitivity analysis (PSA) using 10,000 Monte Carlo simulations in which model inputs were simultaneously varied within their lower and upper ranges using specified distributions as shown in Table 1. Lower and upper ranges were estimated using the 95% CIs of the original sources. The PSA allowed us to estimate 95% credibility intervals (95% CrI) of the burden of HZ using the 2.5 and 97.5 percentiles of the simulations. Additionally, the simulations of the PSA served as an input for a two-way sensitivity analysis, in which the vaccine cost per series of HZ/su and per dose of ZVL were varied at the same time. For each individual simulation, the total costs and QALYs per alternative were converted to net monetary benefits (NMBs) using a cost-effectiveness threshold of €20,000 per QALY gained. Subsequently, for each combination of cost per series or dose, we analysed which alternative had the most simulations with the highest NMB and whether this proportion of simulations for the preferred alternative was above 90% or not.

Additionally, multiple deterministic sensitivity analyses were performed. The annual waning rate of HZ/su after 4 years was varied between 0 and 10% using a time horizon of 15 years and a lifetime time horizon. We also did a one-way sensitivity analysis in which model inputs were varied one-by-one between their lower and upper ranges while keeping other values at their base case levels. Finally, several scenario analyses were performed to explore the impact of different methodological assumptions or inputs from other plausible sources (see scenario column in Table 1 and Additional file 1 for more details). These analyses included the use of epidemiological input estimates from an immunocompetent population; a lower adherence to the second dose of HZ/su of 90%, 70% and 50% relative to the initial dose; and the attribution of a QALY penalty for vaccine-related grade 3 adverse events [4, 11, 19, 20, 49]. For ZVL, we additionally explored the impact of including additional efficacy against PHN as shown in the trial and the use of vaccine effectiveness data from various post-licensure studies [11, 14, 16, 50]. For the latter, we selected three retrospective cohort studies based on the data from the US Kaiser Permanente Northern California database for the period 2007–2015, the US Medicare database for 2007–2014 and UK Clinical Practice Research Datalink database for 2013–2016, and for each study, we fitted a linear model (see Additional file 1 for details).

### Reporting quality and model validation

The reporting of the study adheres to the Consolidated Health Economic Evaluation Reporting Standards (CHEERS) checklist, and the model validation efforts are reported using the Assessment of the ValIdation Status of Health-Economic decision models (AdVISHE) questionnaire [51, 52]. Both checklists are added in Additional files 2 and 3.

## Results

### Current burden of disease

The estimated burden of HZ among the 2017 Dutch population aged ≥ 50 years is presented in Table 2. The total number of HZ cases is estimated at 54,169 (95% CrI 52,130–56,174) per year, resulting in 434 hospital admissions, 805 1-day hospitalizations, 15 deaths and 3298 PHN cases. This generated a QALY loss of 2992 (95% CrI 2418–3771). The major part of the health burden occurred among 60- to 79-year-olds, while most deaths were among ≥ 80-year-olds. The total annual cost burden of HZ was estimated at €19.6 million (95% CrI 16.3–22.6), including €11.3 million healthcare costs and €7.7 million non-healthcare costs. Most healthcare costs were due to GP visits, while non-healthcare costs predominantly consisted of productivity losses among 50- to 69-year-olds.

**Table 2:** Estimated annual burden of herpes zoster in the Netherlands among ≥50-year-olds using January 2017 population data. The 95%-credibility intervals were based on a probabilistic sensitivity analysis using 10,000 Monte-Carlo simulations

	Age group					Total	95% CrI−	95% CrI+
	50–59 years	60–69 years	70–79 years	80–89 years	≥ 90 years			
Population	2,473,222	2,083,983	1,379,744	641,923	121,068	6,699,940		
Health outcomes								
HZ cases	13,160	16,065	14,772	8557	1614	54,169	52,130	56,174
Hospitalizations	61	101	131	118	22	434	382	489
1-day hospital admissions	88	201	301	181	34	805	734	879
HZ deaths	0	0	1	6	7	15	9	21
PHN cases	289	1179	1084	628	118	3298	2474	4118
LYs lost	4	7	16	39	27	93	56	144
QALYs lost	531	941	873	530	116	2992	2418	3771
Costs (€, millions)								
Healthcare costs	2.27	3.50	3.37	1.91	0.23	11.28	9.57	13.43
GP visits, medication, specialist visits	2.08	3.12	2.93	1.70	0.32	10.14	8.49	12.25
Hospitalizations	0.18	0.37	0.48	0.53	0.10	1.66	1.41	1.93
1-day hospital admissions	0.02	0.06	0.08	0.05	0.01	0.23	0.21	0.25
Averted costs due to HZ preterm mortality^a^	− 0.02	− 0.04	− 0.12	− 0.37	− 0.20	− 0.75	− 1.12	− 0.46
Non-healthcare costs	5.02	2.28	0.25	0.14	0.03	7.73	5.85	9.83
Travel costs	0.14	0.20	0.19	0.11	0.02	0.66	0.54	0.80
OTC medication	0.04	0.07	0.06	0.04	0.01	0.22	0.18	0.26
Productivity loss	4.84	2.01	–	–	–	7.44	4.99	11.81
Total costs	7.29	5.78	3.62	2.05	0.26	19.59	16.33	24.62

*CrI* credibility interval, *GP* general practitioner, *HZ* herpes zoster, *LY* life year, *OTC* over-the-counter, *PHN* post-herpetic neuralgia, *QALY* quality-adjusted life year

^a^Averted healthcare costs due to HZ-related preterm mortality were estimated using the life expectancy and annual treatment costs per year from a standardized tool [40]

### Impact of vaccination

The impact of HZ vaccination is presented in Table 3. For each vaccination age, two doses of HZ/su were estimated to prevent considerably more HZ cases as compared to ZVL single dose or ZVL with a booster after 10 years. Depending on age, vaccination with HZ/su at a coverage of 50% would avert 4335–10,896 HZ cases (34.6–37.9% reduction) over the next 15 years, preventing 318–799 PHN cases, 257–600 lost QALYs and €0.93–2.40 million on costs to the society. Vaccination with ZVL would avert 400–4877 HZ cases (3.5–18.0% reduction) and ZVL with a booster 427–6446 HZ cases (3.7–27.5% reduction). In absolute terms, the highest number of cases would be prevented by vaccination of 70-year-olds for HZ/su, while this was 60-year-olds for ZVL and 50-year-olds for ZVL with a booster. As the model is static, the number of prevented HZ cases increases linearly with the coverage rate (Additional file 1: Figure S12). This figure shows that, for instance for 60-year-olds, vaccination with a series of HZ/su at a coverage of 24% would avert an equal amount of HZ cases as vaccination with ZVL at a coverage of 50%.

**Table 3:** Impact, effectiveness and cost-effectiveness of vaccination of Dutch immunocompetent older adults against HZ at a coverage of 50% over a period of 15 years. Vaccination strategies include the herpes zoster subunit vaccine (two doses) or zoster vaccine live (single dose, or single dose + booster after 10 years). Future costs and quality-adjusted life years (QALYs) include an annual discount rate of 4% and 1.5%, respectively

Vaccination strategy	Total HZ cases	HZ cases averted	PHN cases averted	QALYs gained	Total costs saved (€, millions)	NNV to prevent a HZ case	Threshold vaccine cost to equal €20,000 per QALY (€)^a^	Threshold vaccine cost to equal €50,000 per QALY (€)^a^
50 years								
No vaccination	22,613							
ZVL	18,618	3995	118	159.3	2.060	31.7	29.59	67.29
ZVL + booster	16,392	6220	281	268.1	2.777	20.4	27.09	65.51
HZ/su	14,141	8472	324	351.6	4.026	15.0	63.68	146.91
60 years								
No vaccination	27,093							
ZVL	22,215	4877	358	266.9	1.698	22.8	51.37	123.23
ZVL + booster	20,627	6466	474	345.5	2.012	17.2	37.79	95.37
HZ/su	16,833	10,260	753	548.9	3.270	10.9	104.30	252.09
70 years								
No vaccination	31,481							
ZVL	28,368	3113	228	176.9	0.724	34.9	27.48	76.38
ZVL + booster	27,645	3836	281	215.4	0.865	28.3	19.43	58.42
HZ/su	20,585	10,896	799	599.9	2.400	10.0	109.09	274.91
80 years								
No vaccination	11,449							
ZVL	11,050	400	29	24.7	0.092	117.0	0.73	16.56
ZVL + booster	11,022	427	31	26.4	0.095	109.5	− 1.45	11.69
HZ/su	7114	4335	318	256.6	0.930	10.8	106.03	270.59

*HZ* herpes zoster, *HZ/su* herpes zoster subunit vaccine, *NNV* number needed to vaccinate, *PHN* post-herpetic neuralgia, *QALY* quality-adjusted life-year, *ZVL* zoster vaccine live (live-attenuated vaccine)

^a^Cost per series (two doses) of HZ/su or cost per dose of ZVL. Administration costs of €11.36 per dose and travel costs of €0.43 per dose are not included

### Effectiveness and cost-effectiveness of vaccination

Two doses of HZ/su was the most effective strategy for vaccination of 70-year-olds (NNV to prevent a HZ case of 10.0); however, there was little difference as compared to 60- and 80-year-old cohorts (NNVs of 10.9 and 10.8) (Table 3). For ZVL, vaccination age had a considerable impact on the effectiveness against HZ. The most effective age of vaccination was 60 years (NNV of 22.8), but the effectiveness decreased in the older cohorts (NNV of 34.9 for 70 years and 117.0 for 80 years). The effectiveness of ZVL would improve by adding a booster after 10 years, decreasing the NNV to 17.2 for vaccination of 60-year-olds, 28.3 for 70-year-olds and 109.5 for 80-year-olds.

The cost-effectiveness showed a similar pattern as compared to the effectiveness outcomes. For HZ/su, the maximum vaccine cost per series to remain cost-effective to a threshold of €20,000 per QALY gained was highest for vaccination of 70-year-olds, indicating that this would be the optimum age of vaccination from a cost-effectiveness point of view. The threshold vaccine cost per series at this age was estimated at €109.09. For ZVL, the highest threshold vaccine cost per dose was €51.37 for vaccination of 60-year-olds, while adding a booster dose after 10 years resulted in a decrease of the threshold cost per dose to €37.79. Our finding that a ZVL plus a booster had a lower threshold cost per dose as compared to ZVL single dose was also valid for shorter periods between the booster and the first dose, like 5 years. For the vaccination of 80-year-olds, the threshold vaccine cost per dose of ZVL reached slightly above €0. The use of a higher cost-effectiveness threshold of €50,000 per QALY gained increased the maximum vaccine cost allowed considerably. Under this condition, the threshold cost per series of HZ/su for vaccination of 70-year-olds increased to €274.91.

### Sensitivity analyses

**Fig. 1:**
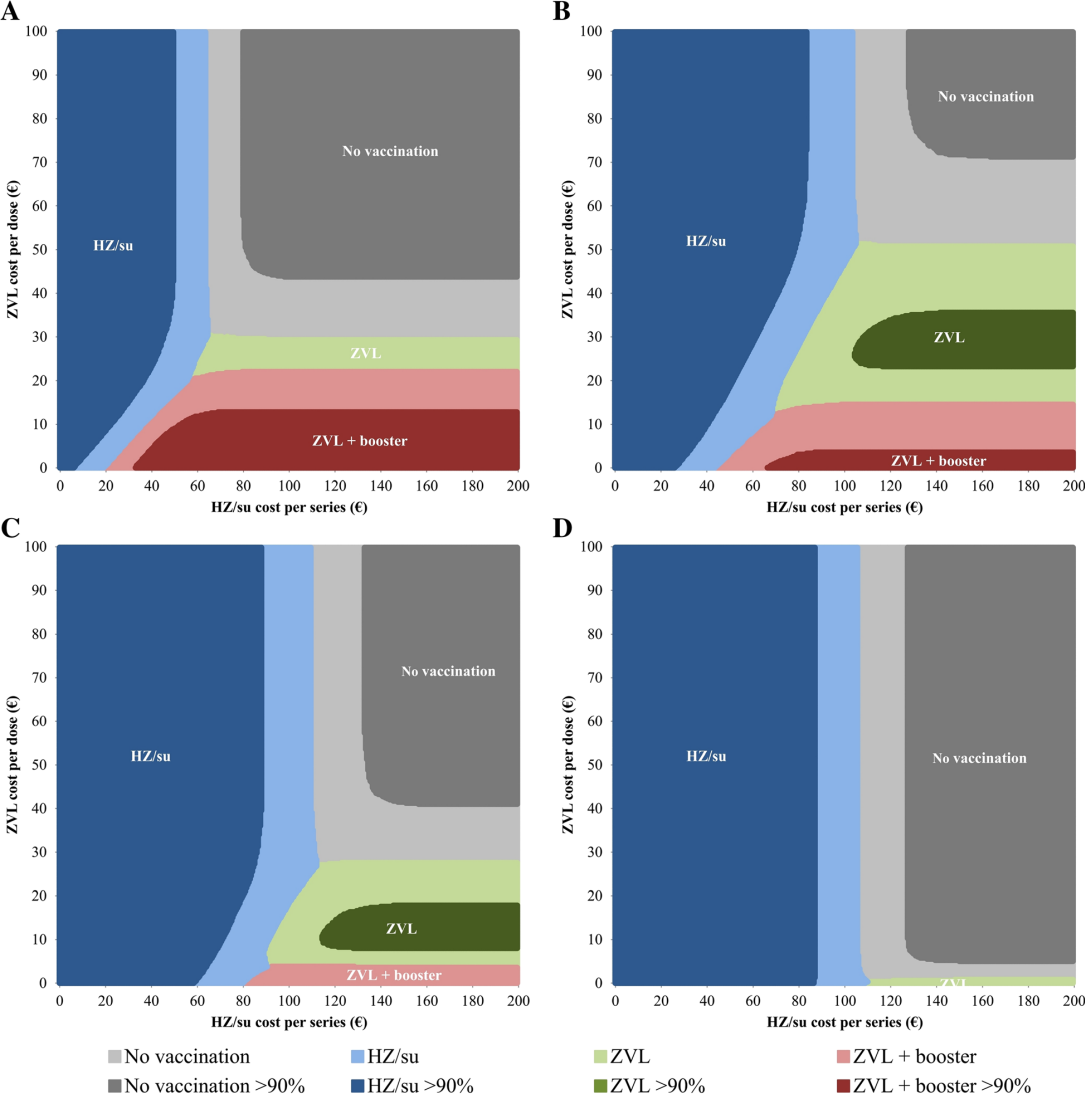
Two-way sensitivity analysis of the vaccine cost per series of HZ/su and vaccine cost per dose of ZVL for vaccination of **a** 50-year-olds, **b** 60-year-olds, **c** 70-year-olds and **d** 80-year-olds. After performing a probabilistic sensitivity analysis using 10,000 Monte Carlo simulations, the alternative with the highest probability of being cost-effective to a willingness-to-pay threshold of €20,000 per QALY gained is presented over a range of vaccine cost. Dark coloured areas indicate that the probability of being the most cost-effective alternative is higher than 90%. HZ/su, herpes zoster subunit vaccine; QALY, quality-adjusted life year; ZVL, zoster vaccine live

A probabilistic two-way sensitivity analysis of the vaccine cost per dose of ZVL versus cost per series of HZ/su is shown in Fig. 1. For each vaccination age, there were combinations of vaccine cost in which either HZ/su, ZVL or ZVL with a booster was the most cost-effective alternative. The competition between HZ/su and ZVL was the highest for 60-year-olds. For instance, if the cost per series of HZ/su was assumed at €100, the maximum cost per dose of ZVL to be the most cost-effective alternative was estimated €46.75. A ZVL booster after 10 years had a higher probability of being cost-effective among 50-year-olds than ZVL single dose when the cost per dose of ZVL was below €21.25. With regard to uncertainty, HZ/su had a more than 90% probability of being the most cost-effective alternative as compared to no vaccination when the vaccine cost per series would fall below €49.74, €85.80, €83.64 and €87.56 for 50-, 60-, 70- and 80-year-olds, respectively.

**Fig. 2:**
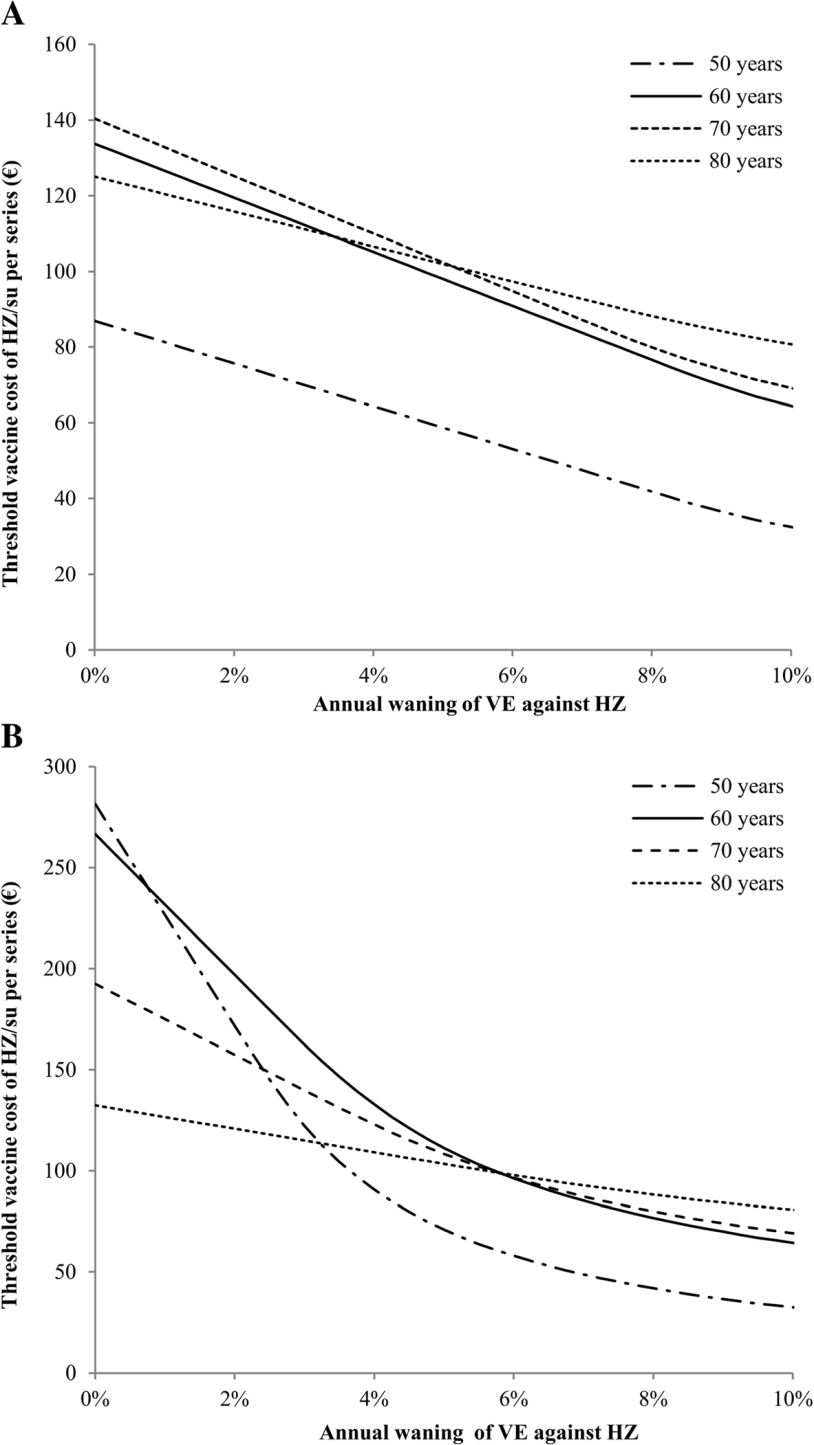
Impact of the annual HZ/su waning rate starting 4 years following vaccination on the threshold vaccine cost per series when using a **a** time horizon of 15 years or **b** a lifetime time horizon. The cost-effectiveness threshold was set at €20,000 per quality-adjusted life year gained. HZ, herpes zoster; HZ/su, herpes zoster subunit vaccine; VE, vaccine efficacy

**Fig. 3:**
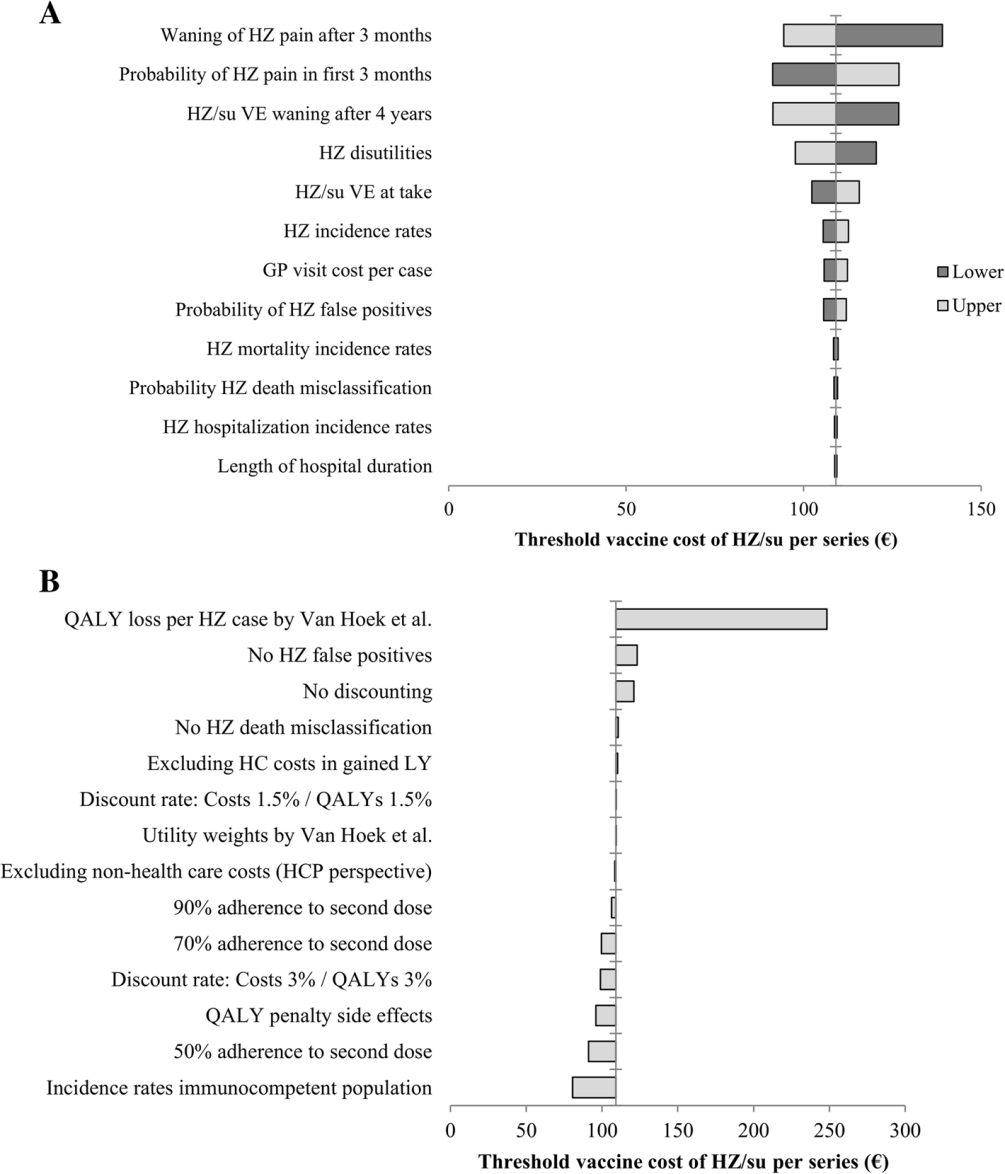
One-way sensitivity analysis of HZ/su for vaccination of 70-year-olds. The threshold vaccine cost per series is the maximum cost to remain below a threshold of €20,000 per QALY gained. **a** Deterministic sensitivity analysis showing the impact of parameter uncertainty by using the lower (dark grey) and upper (light grey) ranges that were based on the 95% confidence intervals of the input parameters. **b** Scenario analysis showing the impact of structural uncertainty by using other plausible model inputs. GP, general practitioner; HC, healthcare; HCP, healthcare payer; HZ, herpes zoster; HZ/su, herpes zoster subunit vaccine; PHN, post-herpetic neuralgia; QALY, quality-adjusted life year; VE, vaccine efficacy

Figure 2 shows the impact of the annual waning rate of HZ/su starting 4 years following vaccination, both when using a time horizon of 15 years (Fig. 2a, base case) and when using a lifetime time horizon (Fig. 2b, sensitivity analysis). Over a period of 15 years, the threshold vaccine cost per series for vaccination of 70-year-olds with HZ/su would change to €140.44 in the absence of waning and to €69.18 when a waning rate of 10% per year was assumed. With regard to the optimum vaccination age, 80 years would become the most cost-effective alternative when the waning rate was higher than 5.0% per year. Using a lifetime time horizon, the threshold cost per series of HZ/su increased to €120.94 for vaccination of 70-year-olds at a waning rate of 4.1% per year as used in the base case analysis. Furthermore, vaccination of 50- and 60-year-olds would become more cost-effective as compared to 70- and 80-year-olds when the waning rate would be lower than 2.3% per year. A sensitivity analysis on the time horizon shows that after 5 years, the threshold vaccine cost was €40.39 per series of HZ/su and €18.88 per dose of ZVL for vaccination of 70-year-olds (Additional file 1: Figure S13). Figure 3 shows a deterministic sensitivity analysis and a scenario analysis for HZ/su at vaccination age of 70 years. The deterministic sensitivity analysis shows that the uncertainty around the waning rate and parameters involved in the estimation of the QALY loss per HZ episode (probability of HZ pain, waning of long-term HZ pain and HZ utilities) had the highest impact on the cost-effectiveness (Fig. 3a). The scenario analysis demonstrates that the threshold vaccine cost per series of HZ/su decreased to €80.54 when HZ incidence estimates from an immunocompetent population were used (Fig. 3b). Reduction of the adherence to the second dose of HZ/su to 90%, 70% and 50% resulted in lower threshold vaccine cost per series of €106.24, €99.54 and €91.05, respectively. The inclusion of a QALY loss due to grade 3 adverse events decreased the threshold vaccine cost per series to €95.93. The highest impact on the cost-effectiveness was found for using QALY loss estimates per HZ case as previously estimated by Van Hoek et al. [31]. In this scenario, the threshold vaccine cost per series of HZ/su increased to €248.38. Other scenarios that altered the threshold vaccine cost by more than 10% included the use of equal discount rates of 4% for costs and effects, no discounting and the exclusion of HZ false positives. Deterministic sensitivity analyses from other age cohorts showed similar results, except that the exclusion of non-healthcare costs resulted in a more than 10% decrease of the threshold vaccine cost per series among 50-year-olds.

**Fig. 4:**
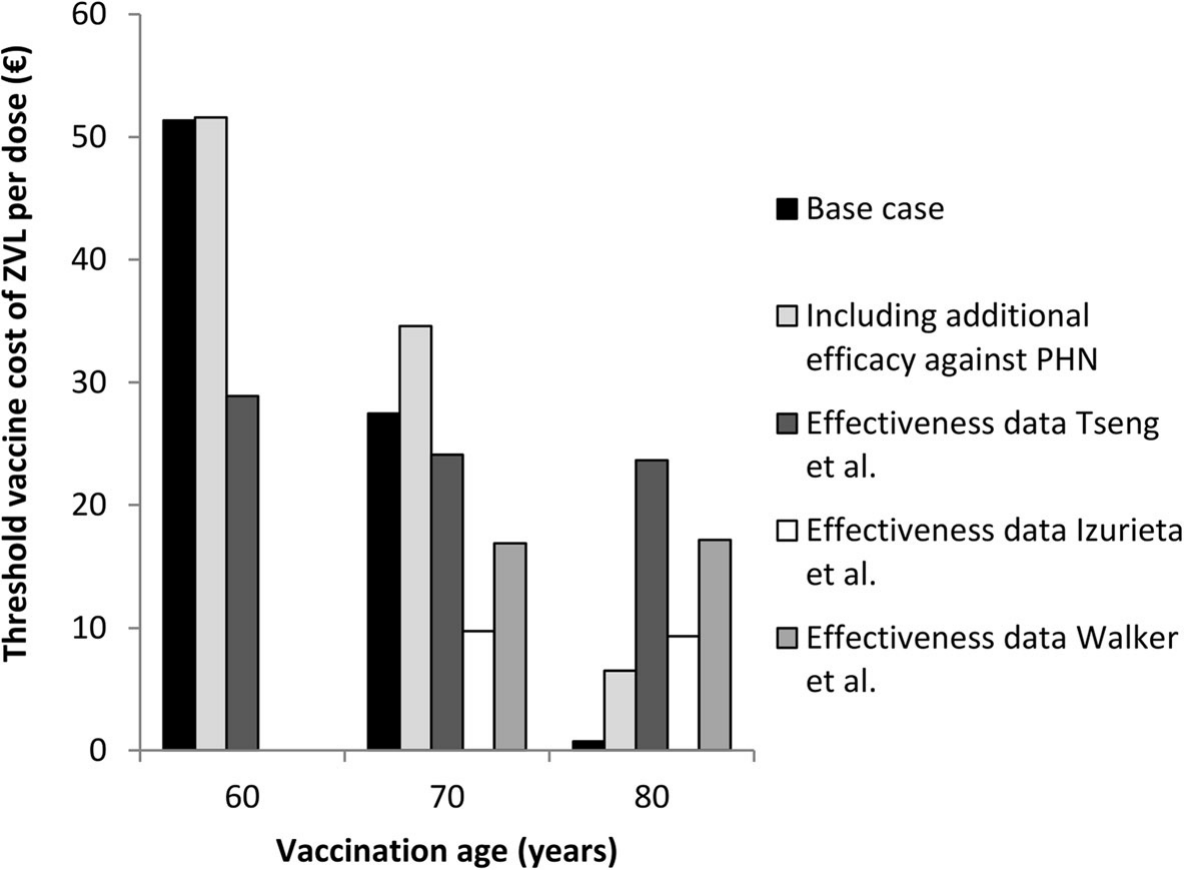
Impact of the inclusion of additional efficacy against PHN using trial data and the use of effectiveness data against HZ on the threshold vaccine cost per dose of ZVL. The cost-effectiveness threshold was set at €20,000 per quality-adjusted life year gained. Effectiveness data from three data sources, i.e. the US Kaiser Permanente Southern California database [14], the US Medicare database [16] and the UK Clinical Practice Research Datalink database [50], were explored. For the Medicare and Clinical Practice Research Datalink database, no effectiveness data of 60-year-olds was available. PHN, post-herpetic neuralgia; QALY, quality-adjusted life year; ZVL, zoster vaccine live

Additional scenarios for ZVL included the consideration of additional efficacy against PHN and the use of post-licensure effectiveness data (Fig. 4). The inclusion of additional efficacy against PHN increased the threshold vaccine cost for 70- and 80-year-olds to €34.56 and €6.52, respectively. The use of ZVL effectiveness data resulted in a decrease of the threshold vaccine cost per dose for 60-year-olds but in a substantial increase for 80-year-olds. For instance, on the basis of 8 years of data from the US Kaiser Permanente Southern California database, the threshold vaccine cost per dose of ZVL for 80-year-olds was estimated at €23.64.

## Discussion

Our analysis demonstrates that vaccination with two doses of HZ/su would result in a substantially higher number of HZ cases prevented and number of QALYs gained as compared to ZVL (single dose or single dose + booster). This was explained by the relatively higher efficacy of HZ/su. However, whether vaccination with HZ/su would be cost-effective as compared to no vaccination or to ZVL depends largely on the vaccine cost per series. We found that especially for the 60-year-old cohort, there are pricing scenarios in which ZVL could potentially be the most cost-effective alternative. Adding a ZVL booster after 10 years was expected to reduce the effectiveness gap between ZVL and HZ/su but required a significant decrease of the vaccine cost per dose to be the most cost-effective alternative. The inclusion of additional efficacy against PHN or the use of recent post-licensure effectiveness data of ZVL was found to improve the cost-effectiveness of ZVL among ≥ 70-year-olds, increasing the competition between the two vaccines in these age cohorts.

### Implications and possible explanations for findings

A threshold of €20,000 per QALY gained is the general Dutch cost-effectiveness threshold for healthcare interventions such as vaccination. At optimum vaccination age, i.e. 70 years for HZ/su and 60 years for ZVL, the maximum vaccine costs to remain cost-effective were estimated at €109 per series and €51 per dose, respectively. These estimates are considerably lower than the currently available vaccine costs of ZVL and HZ/su in the private sector. For instance, the private sector cost of two doses of HZ/su would be €226 (US$280) according to the price list of the Centers for Disease Control and Prevention, and the private sector cost per dose of ZVL in the Netherlands is currently €145 [53, 54]. Our model shows that HZ vaccination would not be cost-effective with either HZ/su or ZVL when the above-mentioned vaccine costs are used.

The optimum age of vaccination with HZ/su would be in the range of 60 to 80 years, as the efficacy is relatively constant with vaccination age. However, the cost-effectiveness and the optimum age of vaccination with HZ/su were highly sensitive to the duration of protection. Currently, the waning rate had to be estimated using 4 years of follow-up data from the trial, showing a decrease in efficacy over time. However, a closer look to the trial data by arm shows that the waning of efficacy within the first 4 years may be caused by a decrease of HZ incidence in the placebo group rather than an increase of HZ incidence in the vaccinated group [20]. Such a decrease of HZ incidence over time in the placebo group is unexpected because the risk of HZ usually increases with age. Our sensitivity analysis demonstrated that a lower waning rate would have a substantial impact on decision-making, as the optimum age of vaccination changed from 60 to 80 years to 50–60 years when a lifetime time horizon was used.

Based on the trial data, the optimum vaccination age of ZVL was 60 years and decreased rapidly with increasing age. However, sensitivity analyses of ZVL demonstrated that the cost-effectiveness of ZVL improves substantially for ≥ 70-year-olds when an additional efficacy against PHN was taken into account. Post-licensure studies also confirmed that ZVL was likely to provide additional protection against PHN as well as to other severe outcomes like ophthalmic HZ and HZ-related hospitalizations [14, 17, 50, 55]. Moreover, effectiveness studies in the US and UK found a relatively stable effectiveness of ZVL with increasing age, explaining why in our sensitivity analysis the cost-effectiveness decreases for 60-year-olds but improves considerably for 80-year-olds when this data was explored in our model. This implies that based on trial data only, the impact of ZVL might be underestimated for the eldest and that ZVL could be a competitor for HZ/su across all age groups. However, it should be noted that although effectiveness studies are more representative for real-life conditions and have more statistical power due to larger sample sizes, they can be affected by uncontrolled confounders. For instance, observational studies rely on the healthcare registries and are therefore more likely to include severe cases as compared to clinical trials that use active surveillance.

Next to cost-effectiveness, budget impact is often considered to be important to decision-makers. As most of the gains are due to the reduction of the burden of illness, vaccination would increase the total healthcare expenditure on HZ considerably. In budget impact analyses, usually a relative short time horizon of maximum 5 years is used and future costs and health effects are not discounted. Under these conditions, the average annual budget impact of vaccination of 50% of the 60-year-olds would be €6.0 million per year for ZVL (one-dose schedule) and €12.7 million per year for HZ/su (two-dose schedule), when corresponding maximum vaccine costs per series of €51 per dose for ZVL and €104 for HZ/su were used. This implies that the implementation of HZ/su would result in a twofold increase of the total healthcare expenditure on HZ as compared to ZVL and a more than fourfold increase as compared to no vaccination.

As HZ/su needs to be administered twice within 2–6 months, vaccination will result in higher healthcare utilization as compared to ZVL that is given as a single dose. Moreover, the prospect of a two-dose regimen might also result in a lower uptake as compared to a one-dose regimen. But we do also note that HZ/su is registered for immunocompromised populations as well, which might be beneficial to the overall vaccination coverage. Both vaccines can be safely combined with influenza vaccination [56, 57], which might facilitate the implementation and save administration costs. However, influenza vaccination is only given once a year, and immunogenicity data indicates that revaccination with HZ/su after 12 months is less immunogenic as compared to 2–6 months after the first dose [58]. Our sensitivity analysis shows that the adherence to the second dose of HZ/su impacts the cost-effectiveness considerably, as a single dose of HZ/su is expected to have a substantially lower efficacy, especially among ≥ 70-year-olds, and a higher waning rate as compared to two doses [47].

Safety studies showed that both HZ/su and ZVL were not associated with serious adverse events among immunocompetent older adults but that HZ/su gives a substantially higher risk of grade 3 adverse events and local adverse events as compared to ZVL [59]. On the other hand, vaccination with HZ/su can also reduce the risk of serious adverse events because post-licensure studies of ZVL indicate that immunocompromised individuals were, although its contraindication, occasionally vaccinated [15, 17]. ZVL can cause serious adverse events in immunocompromised patients, as it may result in a symptomatic, progressive infection of the vaccine virus, causing severe rashes [60].

During the evaluation of ZVL by the Dutch Health Council in 2016, it was concluded that vaccination against HZ did not meet the criteria to be included in the national immunization programme because it does not control VZV transmission nor does it prevent significant mortality [13]. Vaccination against HZ might however be indicated for a public programme when the vaccine would be regarded as essential healthcare due to a substantial reduction of the individual disease burden [61]. The Dutch Health Council considered ZVL not as essential healthcare because of its relatively low efficacy in the eldest, short duration of protection and the contraindication for immunocompromised individuals [13]. Our results demonstrate that HZ/su is expected to have a significantly higher impact on the health economic burden of HZ as compared to ZVL (without or with a booster after 10 years), especially among ≥ 70-year-olds.

In our opinion, these results are also of interest to other countries that are reconsidering HZ vaccination. Recently, the US Advisory Committee on Immunization Practices (ACIP) decided to (i) give HZ/su a preferential status above the ZVL vaccine, (ii) extend the target group from all immunocompetent ≥ 60-year-olds to all immunocompetent ≥ 50-year-olds and (iii) revaccinate individuals that had previously been vaccinated with ZVL [59]. The UK launched a publicly funded vaccination programme using ZVL for 70-year-olds with a catch-up for 78-year-olds in 2013 [62] but now needs to decide whether vaccination with HZ/su should be preferred above ZVL, and if so, whether ZVL-vaccinated individuals should be revaccinated with HZ/su. Since the UK Joint Committee on Vaccination and Immunisation recently suggested a similar cost-effectiveness threshold for vaccines as compared with the Netherlands of £15,000 (€17,400) per QALY gained [63] and the incidence of HZ tends to be similar across European countries [64], the HZ/su threshold vaccine cost per series might be in the same range as we estimated for the Dutch setting. However, the cost-effectiveness of HZ/su in a cohort that is vaccinated with ZVL will presumably be decreased due to a remaining protection offered by ZVL. For instance, with the use of our model, the threshold vaccine cost per series of HZ/su among 70-year-olds decreased from €109.2 per dose to €80.4, €97.6 and €107.2 per series at 1, 3 and 5 years, respectively, after vaccination with ZVL, when using published vaccine effectiveness data from the UK [50].

### Comparison with other studies

A recent study from the US found that the cost-effectiveness of vaccination of 60-year-olds against HZ would remain below a cost-effectiveness threshold of US$50,000 (€40,400) per QALY gained when the vaccine cost per series was below US$360 (€290) for HZ/su and US$350 (€282) for ZVL [21]. These costs were, after adjusting for the higher cost-effectiveness threshold, relatively higher as compared to our findings, which can be explained by the use of a lifetime time horizon, a threefold higher healthcare costs per HZ episode and the inclusion of additional protection against PHN and burden of illness. With the same model, it was demonstrated that a HZ/su booster in individuals previously vaccinated with ZVL would only be cost-effective within 5 years after vaccination if the adherence to the second dose of HZ/su approached 100% [65]. A public health impact study for Germany estimated a similar NNV to prevent a HZ case among ≥ 70-year-olds of 10 for HZ/su and somewhat higher NNV of 50 for ZVL [47]. A cost-effectiveness analysis with the same model found that the ICER of vaccination with HZ/su ranged between €37,000 and €44,000 per QALY gained when the cost per series was €220 [22]. A recent study from Italy estimated the cost-effectiveness of ZVL while taking into account the effect of demographic changes over time and an accompanying varicella vaccination programme [5]. They found that the incidence of HZ is expected to increase over the next decades due to the ageing of the population, that varicella vaccination might cause a further increase of the incidence of HZ because of the reduction of exogenous boosting and that HZ vaccination would cost-effectively reduce this increasing burden of HZ. Finally, two earlier Dutch studies assessing the cost-effectiveness of ZVL estimated ICERs of €22,000 per QALY gained and €30,000 per QALY gained for 70-year-olds using vaccine cost per dose of €77 and €87, respectively [66, 67]. The most important explanation for finding a relatively lower threshold cost per dose in our current study was a substantially lower QALY loss per HZ case. Some other differences were the use of updated HZ incidence rates and long-term efficacy data of ZVL.

### Strengths and limitations

The main strength of our study is that we were able to combine high-quality vaccine efficacy data from large clinical trials with HZ burden estimates from national data sources. HZ incidence was obtained from a GP network that has been validated as a good representation of the general Dutch population. Cost and QALY loss estimates were obtained from a large Dutch prospective cohort study with a long-term follow-up period of 12 months after onset and using the validated EQ-5D instrument to estimate HR-QoL. Moreover, we included a vaccination alternative of ZVL with a booster in our analysis, which has not been compared with HZ/su so far and we are the first exploring post-licensure effectiveness data of ZVL in a cost-effectiveness model.

Our analysis also has its limitations. The duration of protection of HZ/su is currently unknown, which appeared to be an important parameter in the sensitivity analysis. Next, data on adherence to the second dose of HZ/su and the efficacy and waning of one dose of HZ/su is scarce, which also was shown to have an impact on the cost-effectiveness. Also, applying data from the total Dutch population on immunocompetent cohorts might have led to an overestimation of the impact of vaccination. However, we performed a sensitivity analysis by adjusting the epidemiological parameters for this aspect. The patient recruitment and data acquisition of the prospective cohort study that was mainly used for the estimation of HZ-related costs and QALY losses were partly web-based, which might have introduced a selection bias due to the inclusion of healthier subjects able to understand and fill out a web-based questionnaire. However, the target group in our model consisted of immunocompetent individuals, which might also represent a healthier cohort than the general population. Finally, rare HZ-related complications like monaural deafness and monocular blindness were not included in our model.

### Future recommendations

For the future, we recommend an update of our analysis when long-term efficacy data of HZ/su become available. Currently, the long-term duration of protection of HZ/su is investigated in a subpopulation of the trial, while also the impact of revaccination with one or two doses of HZ/su will be assessed [68]. Next, we would recommend cost-effectiveness studies specifically for immunocompromised populations. Recent non-peer-reviewed results of HZ/su for autologous haematopoietic stem cell transplant recipients showed that HZ/su was 68.2% (95% CI 55.6–77.5) efficacious against HZ, while no safety issues occurred [69]. As the risk of HZ among autologous haematopoietic stem cell transplant recipients has been estimated at 16–31% within the first year after transplantation [70], vaccination of such a target group might potentially result in improved cost-effective outcomes as compared to vaccination of immunocompetent individuals only.

## Conclusions

Two doses of HZ/su was found to be superior in reducing the burden of HZ among Dutch immunocompetent older adults as compared to ZVL single dose or ZVL single dose with a booster after 10 years. Vaccination could potentially be cost-effective for both HZ/su and ZVL in the context of the conventional Dutch cost-effectiveness threshold of €20,000 per QALY gained, but this depends largely on the vaccine cost. It is anticipated that these results will be useful for policy-makers in the Netherlands and in all other countries considering HZ vaccination.

## Additional files

Additional file 1: Supplemental methods. (DOCX 567 kb)

Additional file 2: CHEERS checklist. (DOCX 27 kb)

Additional file 3: AdVISHE checklist. (DOCX 42 kb)

## Abbreviations

ACIP: Advisory Committee on Immunization Practices
AdVISHE: Assessment of the ValIdation Status of Health-Economic decision models
CHEERS: Consolidated Health Economic Evaluation Reporting Standards
CI: Confidence interval
EQ-5D-3L: Euroqol-5 dimensions-3 level version
GP: General practitioner
HR-QoL: Health-related quality of life
HZ: Herpes zoster
HZ/su: Herpes zoster subunit vaccine
ICD: International Statistical Classification of Diseases and Related Health Problems
ICER: Incremental cost-effectiveness ratio
LY: Life year
NMB: Net monetary benefit
NNV: Number needed to vaccinate
OTC: Over-the-counter
PHN: Post-herpetic neuralgia
PSA: Probabilistic sensitivity analysis
QALY: Quality-adjusted life year
UK: United Kingdom
US: United States
VZV: Varicella-zoster virus
ZVL: Zoster vaccine live
