# Supplementary material for: Cost-effectiveness of vaccination of immunocompetent older adults against herpes zoster in the Netherlands: a comparison between the adjuvanted subunit and live-attenuated vaccines
Source: BMC Med. 2018 Dec 6;16:228. doi: 10.1186/s12916-018-1213-5 (PMC6282315; doi:10.1186/s12916-018-1213-5)
Supplement: Supplementary file 1 — Supplemental methods. (DOCX 567 kb) [file 12916_2018_1213_MOESM1_ESM.docx]

# Supplemental Methodology

Cost-effectiveness of vaccination of immunocompetent older adults against herpes zoster in the Netherlands: A comparison between the adjuvanted subunit and live-attenuated vaccine

## Health economic decision model


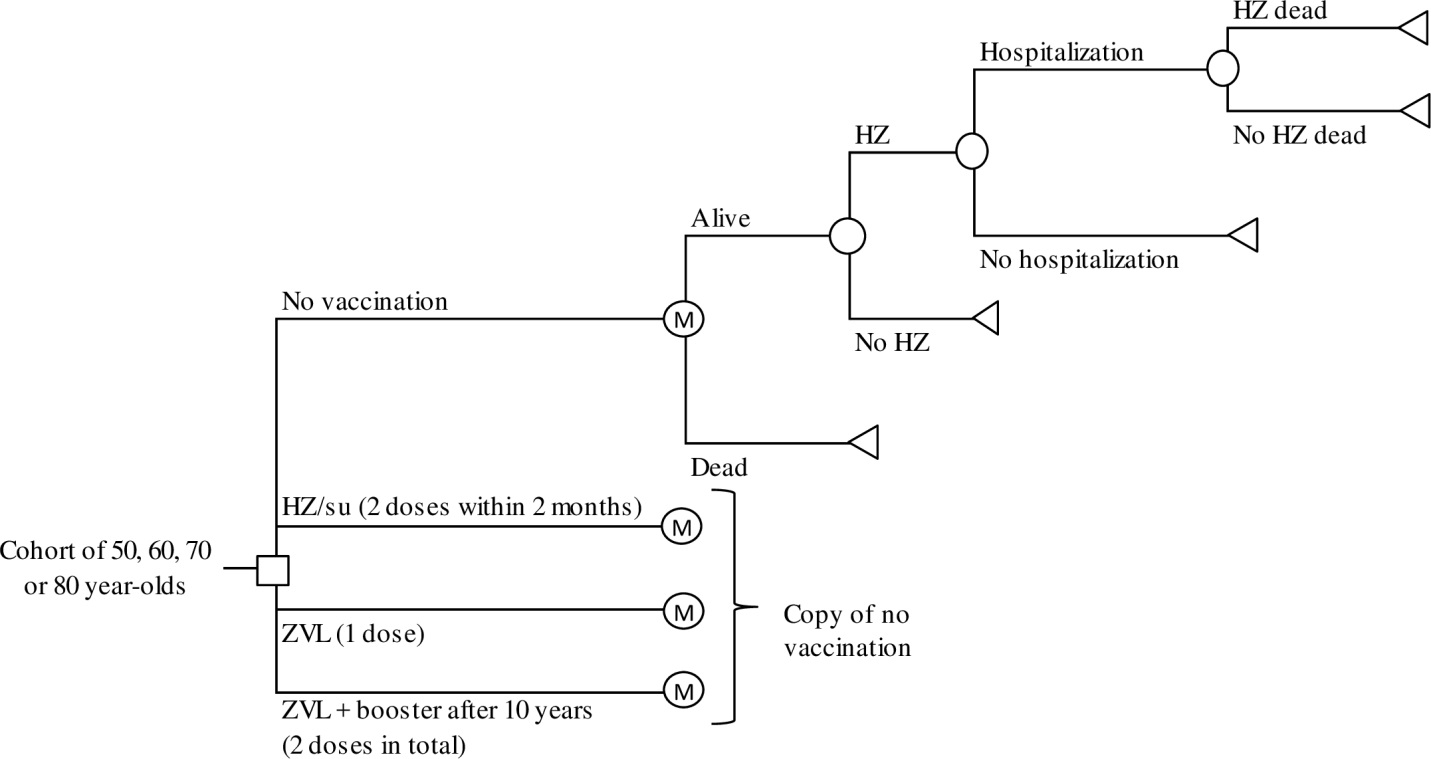


Figure S1: Markov-tree. HZ: Herpes zoster, HZ/su: Herpes zoster subunit vaccine, M: Markov, y: years, ZVL: Zoster vaccine live

## QALY-losses

QALY losses were estimated using a Dutch observational study by Van Wijck et al.[1] that prospectively followed the pain severity and HR-QoL of 661 enrolled patients over a period of 12 months. Loss of follow-up during the study was 8.2% at 6 months and 19.5% at 12 months. At time-points of inclusion, and after 2 weeks and 1, 3, 6, 9 and 12 months, HZ patients were asked to fill in a web-based questionnaire including questions on severity of pain using the VAS-score and the HR-QoL. We categorized the patients into four pain states based on the 10-cm VAS score, which were no pain (VAS <0.5cm), mild pain (VAS 0.5-<3cm), moderate pain (VAS 3-<7cm) and severe pain (VAS ≥7cm). The severity of pain over time was modelled separately for the age-groups 50-59 years and ≥60 year. We used this age-stratification because there was no significant difference of the risk of HZ pain over time between 60-to 69-year-olds and ≥70-year-olds, while a significant difference was found between 50- to 59-year-olds and ≥60-year-olds. The division of HZ patients over the pain states over time is shown in Figure S2. Over the first three months, the division between the pain states was estimated by interpolation between the time-points (Table S1). Patients with moderate or severe pain (i.e. VAS ≥3cm) after three months were defined as PHN patients. For the period after three months, the waning of pain over time for each pain severity state was estimated by fitting an exponential curve through the pain severity data of 3, 6, 9 and 12 months. This curve was fitted on aggregated data of all age-groups because of the low number of patients with moderate or severe pain after a period of 6 months (Figure S3).


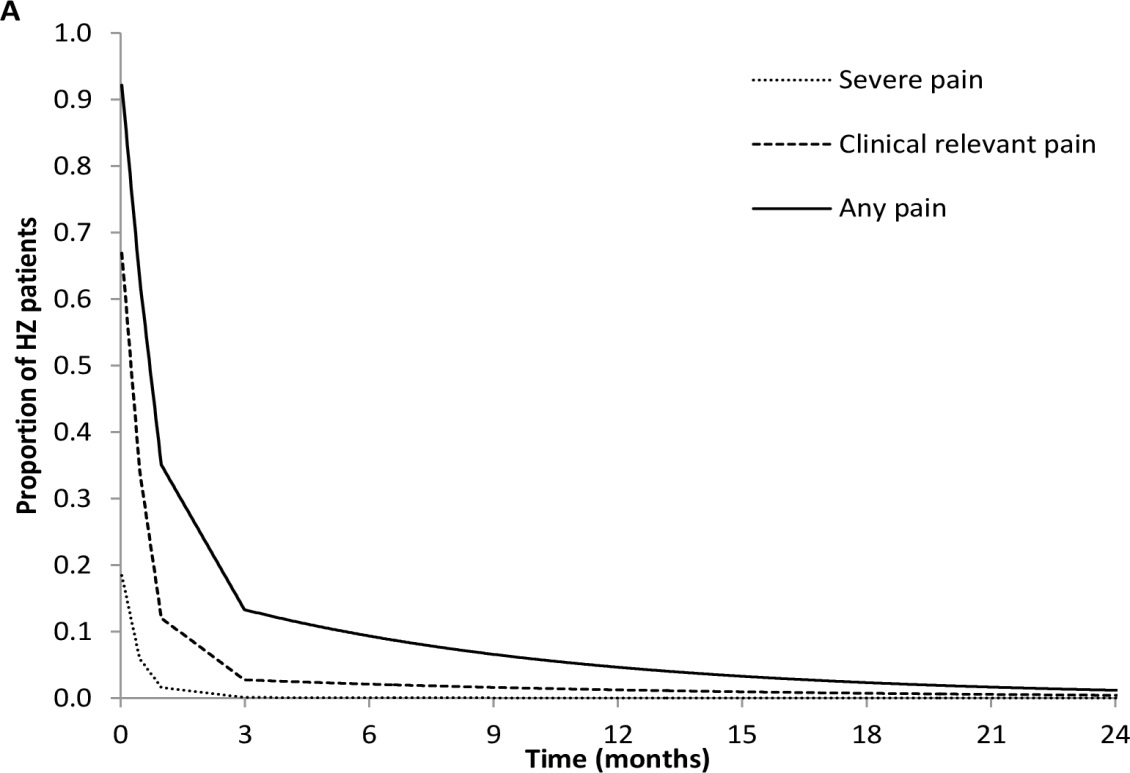


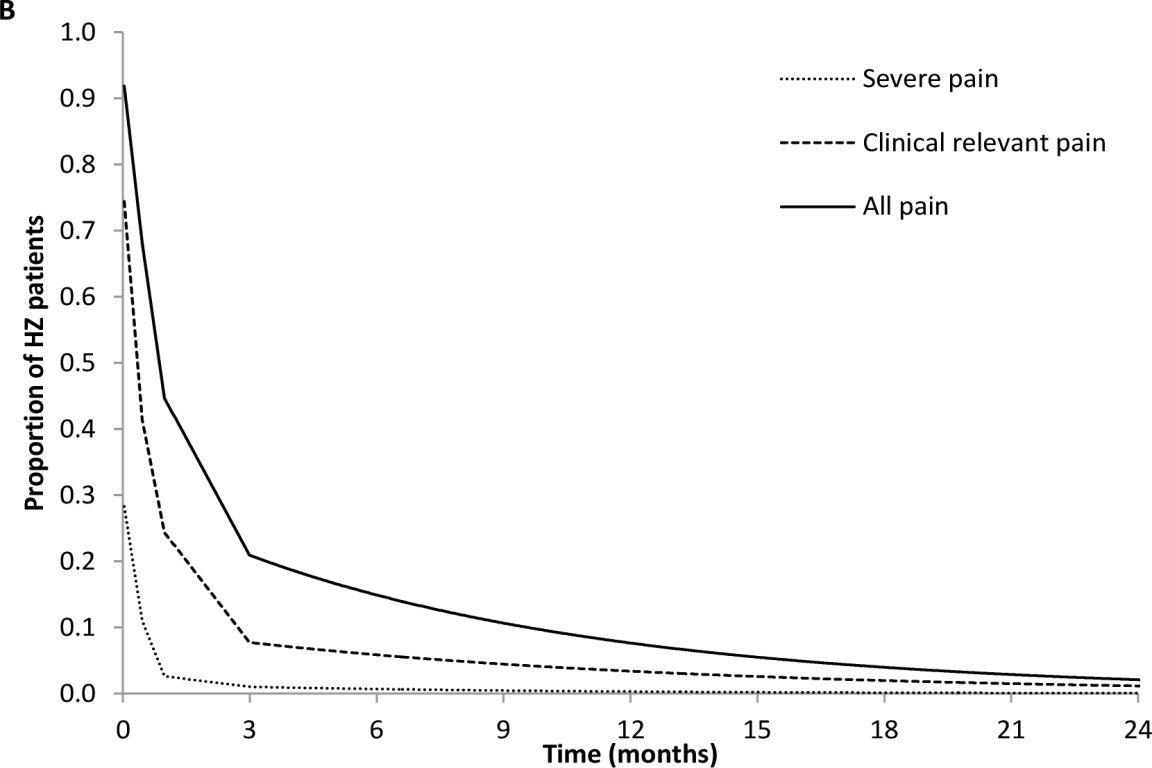


Figure S2: Proportion of herpes zoster (HZ) patients with pain for the age-group by pain severity for the age-group (A) 50-59 years and (B) ≥60 years. Clinical relevant pain includes severe and moderate pain; any pain includes clinical relevant pain and mild pain. Data was obtained from Van Wijck et al.[1]

Table S1: Probability of herpes zoster pain by age and severity over time over the first 3 months after disease onset. Data was obtained from Van Wijck et al.[1]

| **Health state** | **Base case** | **Lower** | **Upper** | **Distribution** |
| --- | --- | --- | --- | --- |
| 50-59 years |  |  |  |  |
| Mild pain |  |  |  |  |
| Inclusion | 0.250 | 0.194 | 0.306 | Beta |
| 2 weeks | 0.289 | 0.226 | 0.351 | Beta |
| 1 month | 0.229 | 0.171 | 0.287 | Beta |
| 3 months | 0.104 | 0.060 | 0.149 | Beta |
| Moderate pain |  |  |  |  |
| Inclusion | 0.500 | 0.436 | 0.564 | Beta |
| 2 weeks | 0.284 | 0.221 | 0.346 | Beta |
| 1 month | 0.100 | 0.058 | 0.141 | Beta |
| 3 months | 0.022 | 0.001 | 0.043 | Beta |
| Severe pain |  |  |  |  |
| Inclusion | 0.194 | 0.143 | 0.245 | Beta |
| 2 weeks | 0.060 | 0.027 | 0.092 | Beta |
| 1 month | 0.015 | 0 | 0.032 | Beta |
| 3 months | - | - | - | Beta |
| ≥60 years |  |  |  |  |
| Mild pain |  |  |  |  |
| Inclusion | 0.168 | 0.132 | 0.203 | Beta |
| 2 weeks | 0.267 | 0.223 | 0.311 | Beta |
| 1 month | 0.202 | 0.161 | 0.242 | Beta |
| 3 months | 0.130 | 0.096 | 0.165 | Beta |
| Moderate pain |  |  |  |  |
| Inclusion | 0.473 | 0.426 | 0.520 | Beta |
| 2 weeks | 0.304 | 0.258 | 0.350 | Beta |
| 1 month | 0.215 | 0.173 | 0.256 | Beta |
| 3 months | 0.065 | 0.040 | 0.090 | Beta |
| Severe pain |  |  |  |  |
| Inclusion | 0.296 | 0.253 | 0.339 |  |
| 2 weeks | 0.110 | 0.079 | 0.141 | Beta |
| 1 month | 0.024 | 0.008 | 0.039 | Beta |
| 3 months | 0.008 | 0 | 0.017 | Beta |


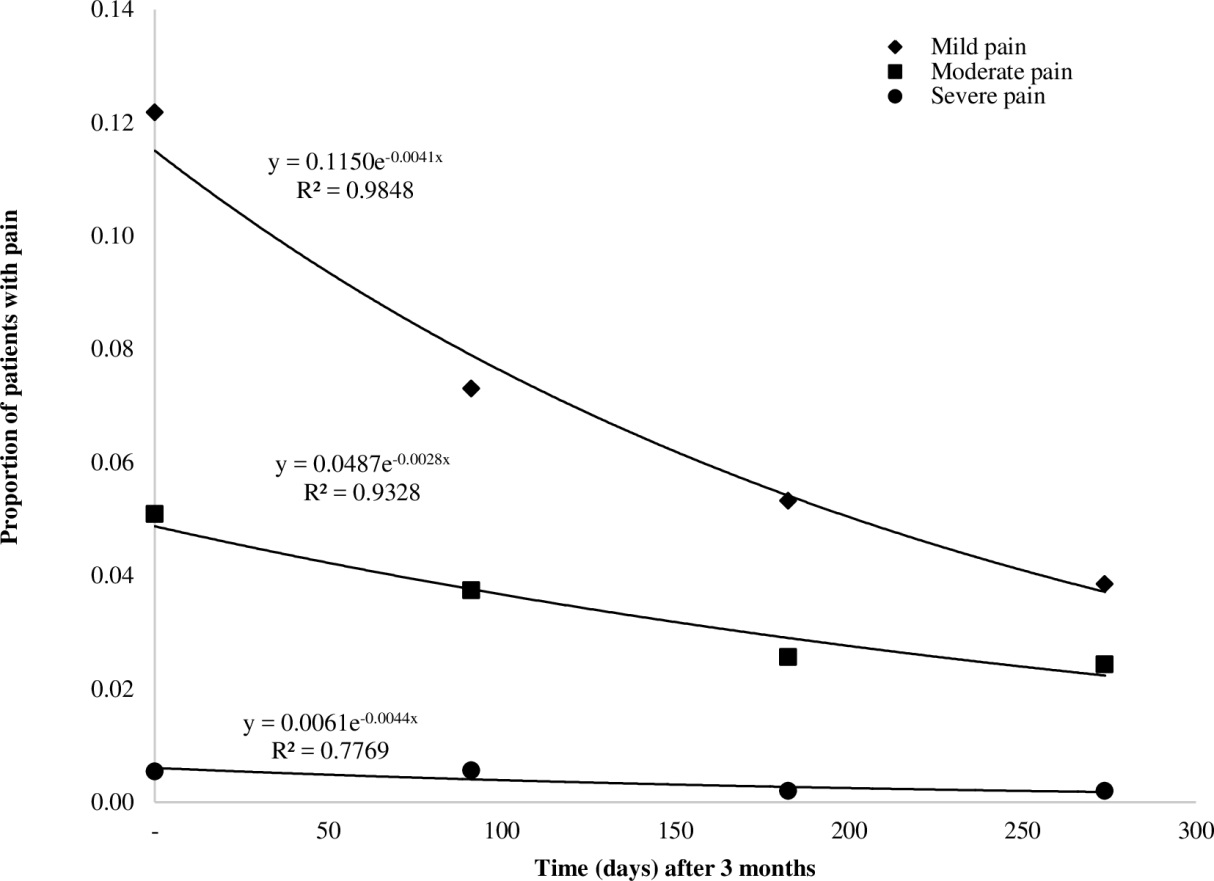


Figure S3: Modelled exponential decline of pain due to HZ over time after a period of 3 months for patients aged ≥50 years. Data was obtained from Van Wijck et al.[1]

HR-QoL data over time was collected with use of the EQ-5D-3L instrument. The EQ-5D-3L is a combination of a questionnaire and a valuation technique using VAS. This instrument values health-related quality of life in five dimensions, i.e. mobility, self-care, usual activities, pain/discomfort and anxiety/depression, using three levels: no problems, some problems and severe problems. As a baseline of HR-QoL we used data from participants that were free of pain or itch after ≥3 months of disease onset. Disutilities were then calculated by subtracting the HR-QoL of the pain state from this baseline. We calculated average EQ-5D-3L scores per pain-severity level at inclusion, after 2 weeks and after 1 month. For the period of 3 months and after, we used aggregated EQ-5D-3L scores from the 3, 6, 9 and 12 months measurement points due to low patient counts. EQ-5D-3L scores are shown in Table S2 and Figure S4.

Table S2: Utilities of the different pain severity states of herpes zoster over time using EQ-5D-3L data. Data was obtained from Van Wijck et al.[1]

| **Pain severity state by measurement point** | **Average  EQ-5D-3L score** | **Lower** | **Upper** | **Distribution** |
| --- | --- | --- | --- | --- |
| Baseline | 0.967 | 0.963 | 0.972 | Beta |
| No pain |  |  |  |  |
| Inclusion | 0.910 | 0.861 | 0.959 | Beta |
| 2 weeks | 0.951 | 0.936 | 0.967 | Beta |
| 1 month | 0.959 | 0.949 | 0.970 | Beta |
| Mild pain |  |  |  |  |
| Inclusion | 0.782 | 0.754 | 0.810 | Beta |
| 2 weeks | 0.786 | 0.764 | 0.808 | Beta |
| 1 month | 0.798 | 0.785 | 0.812 | Beta |
| ≥ 3 months | 0.772 | 0.756 | 0.788 | Beta |
| Moderate pain |  |  |  |  |
| Inclusion | 0.705 | 0.684 | 0.727 | Beta |
| 2 weeks | 0.714 | 0.685 | 0.742 | Beta |
| 1 month | 0.760 | 0.732 | 0.788 | Beta |
| ≥ 3 months | 0.754 | 0.730 | 0.778 | Beta |
| Severe pain |  |  |  |  |
| Inclusion | 0.492 | 0.449 | 0.534 | Beta |
| 2 weeks | 0.656 | 0.597 | 0.715 | Beta |
| 1 month | 0.649 | 0.535 | 0.763 | Beta |
| ≥ 3 months | 0.600 | 0.495 | 0.705 | Beta |


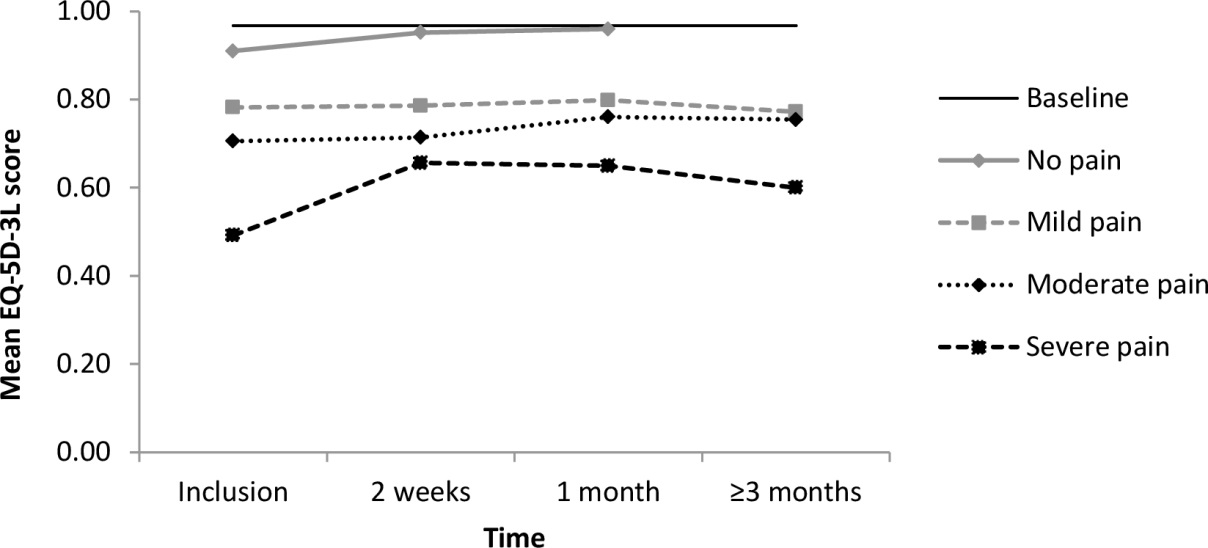


Figure S4: Mean EQ-5D-3L score related to herpes zoster by pain severity level. Data was obtained from Van Wijck et al.[1]

LY lost due to HZ-related mortality were estimated using life tables and age-specific life-expectancies from the general population [2]. Life-expectancies were then converted to QALYs using EQ-5D-3L population norms from the general population [3] ( Table S3).

Table S3: EQ-5D-3L population norms by age-group

| Age-group | EQ-5D-3L population norm |
| --- | --- |
| 50-54 years | 0.890 |
| 55-64 years | 0.890 |
| 65-74 years | 0.886 |
| ≥75 years | 0.830 |

## Costs

HZ-related costs were calculated by multiplying the HZ-related resource use with the price per resource unit. The following cost-items were included in our analysis:

- Health care costs: Costs due to vaccine administration, general practitioner (GP) visits, prescribed medication, specialist visits, one-day hospital admissions, hospitalizations and health-care costs in gained life years.
- Patient costs: Costs due to over-the-counter (OTC) medication and travelling.
- Productivity losses: Costs due to work absenteeism and work presenteeism.

HZ-related resource use is shown in Table S3. The number of GP visits, specialist visits and medication use were estimated using the aforementioned prospective cohort study by Van Wijck et al.[1] Since we based our HZ incidence on the surveillance of HZ-related GP visits, we assumed that every HZ case would have at least one GP visit. The number of follow-up visits was estimated using data up to 6 months after HZ onset finding on average 0.88 additional visits. However, as more GP visits would presumably follow after the period of 6 months among patients with pain, we assumed that the number of GP visits were equally distributed over the 6 months, resulting in an average of 0.15 per month (0.88 / 6 months). Assuming that only patients with pain had GP visits, these visits were assigned to 14.7% of the cohort that had any pain between 3 and 6 months, resulting in 0.15/0.147 = 1.0 GP visits per month given pain after 3 months. This was multiplied by the average duration of any pain by age-group given any pain at 3 months, which was estimated at 13.3 months for 50- to 59-year-olds and 14.1 months for ≥60-year-olds (based on the duration of pain estimation as shown in Figure S4). The probability of a referral to the medical specialist was 6.0% (95% CI: 4.1-7.8%). Based on expert opinion we assumed that each referred patient had on average three specialist visits. The probability and type of medication used was based on self-reporting of patients over a period of 12 months. Length of stay of a hospital admission by age was obtained from the Dutch Hospital Database, using admissions over the period 2012-2014 with HZ as the main diagnose (ICD-code B02) [4].

Productivity loss per HZ case was also obtained from the prospective cohort study by Van Wijck et al.[1] A standardized questionnaire was used to measure productivity losses at HZ onset, and after 3 and 6 months [5]. Patients were asked whether they had absenteeism due to medical conditions over the last month and the number of days missed. Notably, this could also include absenteeism that was not related to HZ. The data showed that the mean absenteeism over the last month was 2.87 days (47.7% of the patients reported absenteeism of on average 6.02 days) at disease onset, 0.92 days (15.1%, 6.09 days) after 3 months of onset and 0.80 (10.1%, 7.89 days) after 6 months of onset. We adjusted these estimates for background absenteeism using data from Statistics Netherlands, showing that the average absenteeism per employee aged 50-69 years was 0.80 days per month [6]. Subtracting the background absenteeism from the reported absenteeism of the cohort, we estimated an absenteeism of 2.07 days over the last month at disease onset, 0.12 days over the last month after 3 months and 0 days over the last month after 6 months. We assumed that only patients with pain had absenteeism, and the total number of days per HZ case were estimated by multiplying the proportion of patients with pain over a month by the days lost per month over time. Finally, the number of days lost were adjusted for age-specific labour participation rates [7].

Occurrence and duration of presenteeism was enquired in the same questionnaire by asking the number of hours that were required to repair missed work due to medical conditions over the last month. The mean presenteeism-related work-loss was 5.68 hours (70% of the patients reported presenteeism of on average 8.14 hours) at onset, 0.45 hours (24%, 1.83 hours) at 3 months and 0.09 hours (12%, 0.73 hours) at 6 months. Based on the average number of workhours per day of 5.93 for 50- to 69-year-olds, these numbers were converted to 0.96, 0.08 and 0.01 days, respectively. No background presenteeism is available from Statistics Netherlands, however, the ratio of hours missed due to presenteeism and absenteeism has been estimated previously at 0.32 (data from 2008) [8]. This would result in a background presenteeism of 0.80 days x 0.32 = 0.25 days per month. Subtracting the background presenteeism from the presenteeism of the cohort study, we found a presenteeism of 0.70 days at HZ onset, while presenteeism after 3 and 6 months became below zero and was therefore ignored. Total presenteeism per case was calculated with similar methods that were used for estimating the absenteeism per case. Finally, productivity losses due to HZ-related preterm mortality were estimated using the friction-cost method, assuming that productivity losses are limited to a friction period of 84 days, because an employee is assumed to be replaced afterwards [9, 10].

Table S4: HZ-related resource use

| **Variable** | **Base case** | **Lower** | **Upper** | **Distribution** | **Ref.** |
| --- | --- | --- | --- | --- | --- |
| Number of GP visits per HZ case |  |  |  |  | Van Wijck, 2016 [1] |
| 50-59 years | 3.00 | 2.72 | 3.28 | Gamma |  |
| ≥60 years | 4.01 | 3.58 | 4.43 | Gamma |  |
| Proportion specialist visit | 0.060 | 0.041 | 0.078 | Beta | Van Wijck, 2016 [1] |
| Number of specialist visits given referral | 3 |  |  |  | Assumption |
| Probability of medication prescription |  |  |  |  | Van Wijck, 2016 [1] |
| Antiviral drugs |  |  |  |  |  |
| Aciclovir | 0.110 | 0.086 | 0.135 | Beta |  |
| Famciclovir | 0.048 | 0.032 | 0.065 | Beta |  |
| Valaciclovir | 0.277 | 0.237 | 0.317 | Beta |  |
| Opiates |  |  |  |  |  |
| Codeine / paracetamol | 0.033 | 0.020 | 0.047 | Beta |  |
| Tramadol | 0.091 | 0.070 | 0.111 | Beta |  |
| Oxycodone | 0.014 | 0.005 | 0.022 | Beta |  |
| Morphine | 0.012 | 0.004 | 0.020 | Beta |  |
| Fentanyl | 0.009 | 0.003 | 0.015 | Beta |  |
| Topical treatment |  |  |  |  |  |
| Hydrocortisone | 0.009 | 0.002 | 0.016 | Beta |  |
| Capsaicin | 0.005 | - | 0.010 | Beta |  |
| Antiepileptic drugs |  |  |  |  |  |
| Pregabalin | 0.071 | 0.051 | 0.091 | Beta |  |
| Carbamazepine | 0.002 | - | 0.004 | Beta |  |
| Gabapentin | 0.006 | 0.000 | 0.012 | Beta |  |
| Tricyclic antidepressant |  |  |  |  |  |
| Amitriptyline | 0.064 | 0.044 | 0.083 | Beta |  |
| Nortriptyline | 0.005 | - | 0.010 | Beta |  |
| Length of stay hospitalization (days) |  |  |  |  | Dutch Hospital Data, 2012-2014 [4] |
| 50-59 years | 5.9 | 5.1 | 6.6 | Gamma |  |
| 60-69 years | 7.5 | 6.5 | 8.4 | Gamma |  |
| 70-79 years | 7.5 | 6.8 | 8.2 | Gamma |  |
| ≥80 years | 9.2 | 8.4 | 10.1 | Gamma |  |
| Probability of OTC medication |  |  |  |  | Van Wijck, 2016 [1] |
| Pain killers |  |  |  |  |  |
| Paracetamol | 0.383 | 0.023 | 0.337 | Beta |  |
| NSAID | 0.171 | 0.016 | 0.139 | Beta |  |
| Topical |  |  |  |  |  |
| Zink oxide | 0.050 | 0.009 | 0.033 | Beta |  |
| Lidocaine | 0.077 | 0.011 | 0.056 | Beta |  |
| Hydrophobic ointment | 0.085 | 0.011 | 0.063 | Beta |  |
| Absenteeism given HZ (days) |  |  |  |  | Van Wijck, 2016 [1], adjusted for age-specific labour participation rates. People aged ≥70 years were assumed to be retired |
| 50-59 years | 1.72 | 0.99 | 3.21 | Gamma |  |
| 60-69 years | 0.76 | 0.43 | 1.45 | Gamma |  |
| ≥70 years | 0 |  |  |  |  |
| Presenteeism given HZ (days) |  |  |  |  |  |
| 50-59 years | 0.54 | 0.30 | 0.81 | Gamma |  |
| 60-69 years | 0.24 | 0.13 | 0.36 | Gamma |  |
| ≥70 years | 0 |  |  |  |  |
| Number of working days lost HZ death | 84 |  |  |  | Friction period [10] |

HZ: Herpes zoster, GP: General practitioner, NSAID: Nonsteroidal anti-inflammatory drug

Costs per unit are based on standardized costing data and are listed in Table S4. Drug regimen schemes were assumed to be equal to standard available Dutch treatment guidelines for neurological pain [11]. Duration of short-term treatment was assumed to be 7 days for antivirals and 1 month for topical medication. Pain treatment was assumed to be equal to the average duration of any pain like used for estimating the number of GP visits. Additionally, prescription only drugs included a €12 pharmacy fee, consisting of a €6 standard prescription fee and a €6 fee for instructions at first time use [10]. Since the most recent update of the Dutch guidelines for cost-effectiveness research, healthcare costs in gained life-years of averted deaths, i.e. indirect healthcare costs, should be taken into account [10]. We estimated these costs by using age-specific life-expectancy and health care costs per year that were not related to HZ (obtained from the Practical Application to Include Disease Costs (PAID) toolkit [12]). Travel costs included transportation costs to the hospital, GP visit, pharmacist and were calculated using Dutch average distances from the specific health care provider multiplied by standard costs of €0.14 per kilometre [10]. Productivity loss per hour was set at €34.75 [10] and was adjusted for the proportion of the population working using age-specific labour participation rates [7]. For ≥70-year-olds we ignored productivity losses, because these people were assumed to be retired.

Table S5: Unit costs (price year 2017)

| **Unit** | **Cost per unit (€)** | **Reference** |
| --- | --- | --- |
| **Health care costs** |  |  |
| GP visit | 33.76 | NHCI, 2016 [10] |
| Specialist visit | 93.11 | NHCI, 2016 [10] |
| Prescribed medication |  | Medicijnkosten.nl, 2017 and Farmacotherapeutisch kompas, 2017 [11, 13] |
| Antiviral drugs |  |  |
| Aciclovir | 34.40 |  |
| Famciclovir | 136.11 |  |
| Valaciclovir | 17.46 |  |
| Opiates |  |  |
| Codeine / paracetamol | 20.70 |  |
| Tramadol | 15.60 |  |
| Oxycodone | 14.82 |  |
| Morphine | 28.80 |  |
| Fentanyl | 16.83 |  |
| Topical treatment |  |  |
| Hydrocortisone | 13.38 |  |
| Capsaicin | 13.23 |  |
| Antiepileptic drugs |  |  |
| Pregabalin | 123.60 |  |
| Carbamazepine | 20.46 |  |
| Gabapentin | 20.46 |  |
| Tricyclic antidepressant |  |  |
| Amitriptyline | 18.12 |  |
| Nortriptyline | 40.08 |  |
| Hospitalization (per day) | 487.01 | NHCI, 2016 [10] |
| Hospital day visit | 282.25 | NHCI, 2009 |
| **Non-healthcare costs** |  |  |
| Travel costs GP visit | 0.43 | NHCI, 2016 [10] |
| Travel costs pharmacist | 0.51 | NHCI, 2016 [10] |
| Travel costs hospital visit | 5.79 | NHCI, 2016 [10] |
| OTC medication |  | Medicijnkosten.nl, 2017[13] and Farmacotherapeutisch kompas, 2017[11] |
| Pain killers |  |  |
| Paracetamol | 10.35 |  |
| NSAID | 15.39 |  |
| Topical |  |  |
| Zink oxide | 4.35 |  |
| Lidocaine | 0.37 |  |
| Hydrophobic ointment | 1.44 |  |
| Productivity loss per lost labour day |  | Productivity loss per hour from NHCI 2016[10] multiplied by the average duration of a work day from Statistics Netherlands, 2016 [7]. |
| 50-59 years | 231.73 |  |
| ≥60 years | 180.02 |  |

GP: General practitioner, OTC: Over-the-counter, NHCI: National Health Care Institute, NSAID: Nonsteroidal anti-inflammatory drug

## Vaccine characteristics

The efficacy of HZ/su against HZ incidence was obtained from two published clinical trials, conducted among individuals aged ≥50 years (ZOE-50 trial) with mean follow-up of 3.2 years and ≥70 years (ZOE-70 trial) with a mean follow-up period of 3.7 years [14, 15]. For the ≥70-year-olds, we fitted a linear model over the annual vaccine efficacy data using R (http://www.R-project.org), in which a weighting factor for the standard error was included. The standard error of the vaccine efficacy by year was estimated as follows: (95%CI_UPPER_ - 95%CI_LOWER_) / (2*1.96). The weighting factor was then calculated by doing 1/(standard error)^2^, meaning that years with a small confidence interval were given a heavier weight. We also tried to fit elbow-shaped functions (exponential, logarithmic) and a knee-shaped function (1-minus-exponential), but, due to the short follow-up period of the data, these models resulted in unrealistic long (~lifelong >95% efficacy) or short durations of protection (to 0% efficacy within 5 years). For the 50- to 69-year-olds, only annual point-estimates without confidence intervals were available [16]. Therefore, we used an unweighted linear model for this age-group. The model fits are shown in Figure S5 and Figure S6.


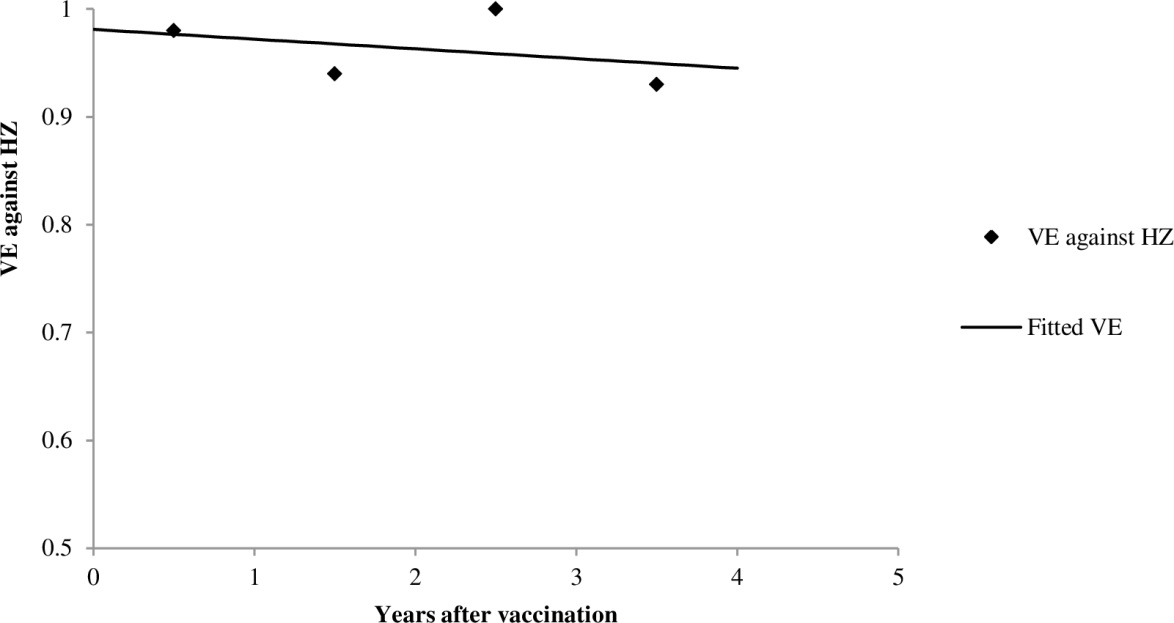


Figure S5: The linear function that was fitted for the herpes zoster subunit (HZ/su) vaccine for 50- to 69-year-olds using trial data up to 4 years. The function was estimated at 0.981 (±0.038) – 0.009 (±0.0169)*years, with R^2^ = 0.124. Note that the x-axis starts at 0.5. HZ: Herpes zoster, VE: Vaccine efficacy.


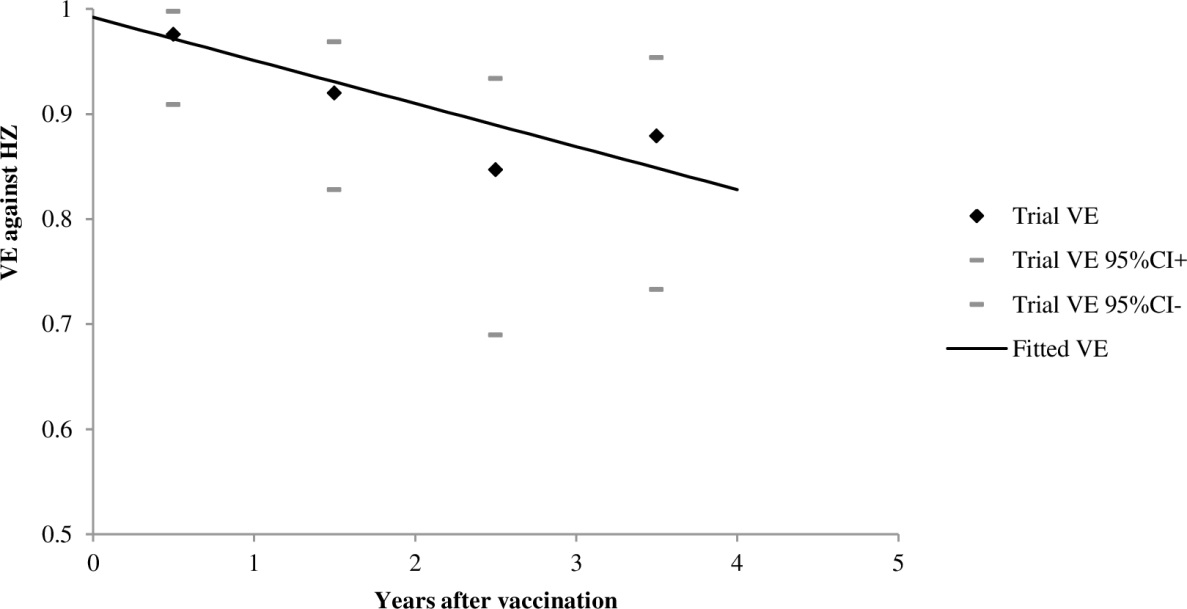


Figure S6: The linear function that was fitted for the herpes zoster subunit (HZ/su) vaccine for ≥70-year-olds using trial data up to 4 years. The function was estimated at 0.992 (±0.018) – 0.041 (±0.012)*years, with R^2^ = 0.857. Note that the x-axis starts at 0.5. HZ: Herpes zoster, VE: Vaccine efficacy.

Efficacy of ZVL over time was modelled using data from published clinical trials. The Shingles Prevention Study (SPS) reported efficacy of ZVL among immunocompetent ≥60-year-olds through 4 years, with a mean follow-up of 3.1 years [17]. After the SPS, a subset of the participants were enrolled in the STPS, which reported efficacy from 3.3 to 7.8 years post-vaccination [18]. The LTPS further assessed the duration of efficacy by following a cohort of SPS vaccine recipients from 4.7 to as long as 11.6 years post-vaccination [19]. As no placebo control was available in the LTPS study, the efficacy for this period was estimated using a historical control group. A one-minus-exponential function was fitted on the annual vaccine efficacy data for ≥60-year-olds with the standard error as a weighting factor. This type of function provided a better fit as compared with a linear function or other knee-shaped and elbow-shaped functions. The one-minus-exponential function is shown in Figure S7.


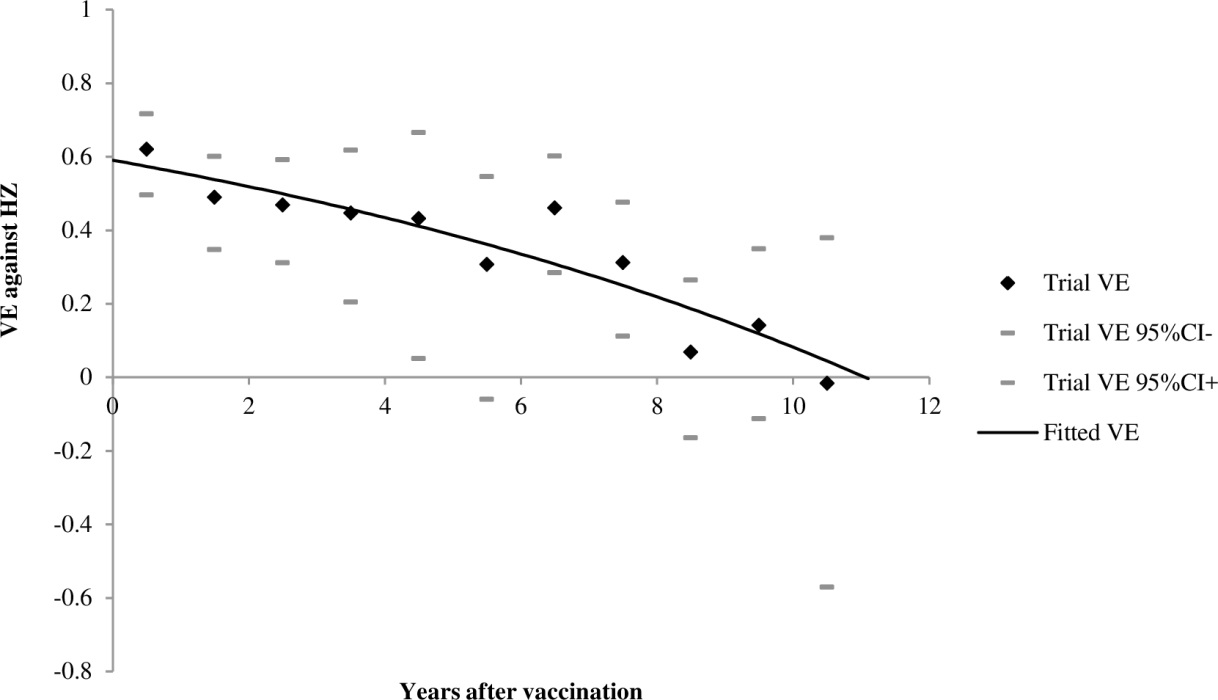


Figure S7: The one-minus-exponential function that was fitted for the live-attenuated vaccine (zoster vaccine live [ZVL]) using trial data up to 11 years [19]. The function was estimated at 1- exp(-0.893 (±0.074) – 0.081 (±0.012)*years), with R^2^ = 0.840. HZ: Herpes zoster, VE: Vaccine efficacy.

The SPS study showed a decline of vaccine efficacy by increasing age. Therefore, we adjusted the vaccine efficacy per age-group using age-specific risk ratios (see main document). Risk ratios were estimated by dividing the age-specific vaccine efficacy estimate of the SPS by the aggregated efficacy. Intercepts were multiplied by the age-specific risk ratio. The risk ratio of 50- to 59-year-olds was calculated using data from an additional trial performed in this age-group [20]. However, as the follow-up period of the trial was only 1.3 years, we first extrapolated the efficacy estimate to 3.1 years using the waning function that we estimated for ≥60-year-olds. We assumed that all age-groups had the same waning rate.


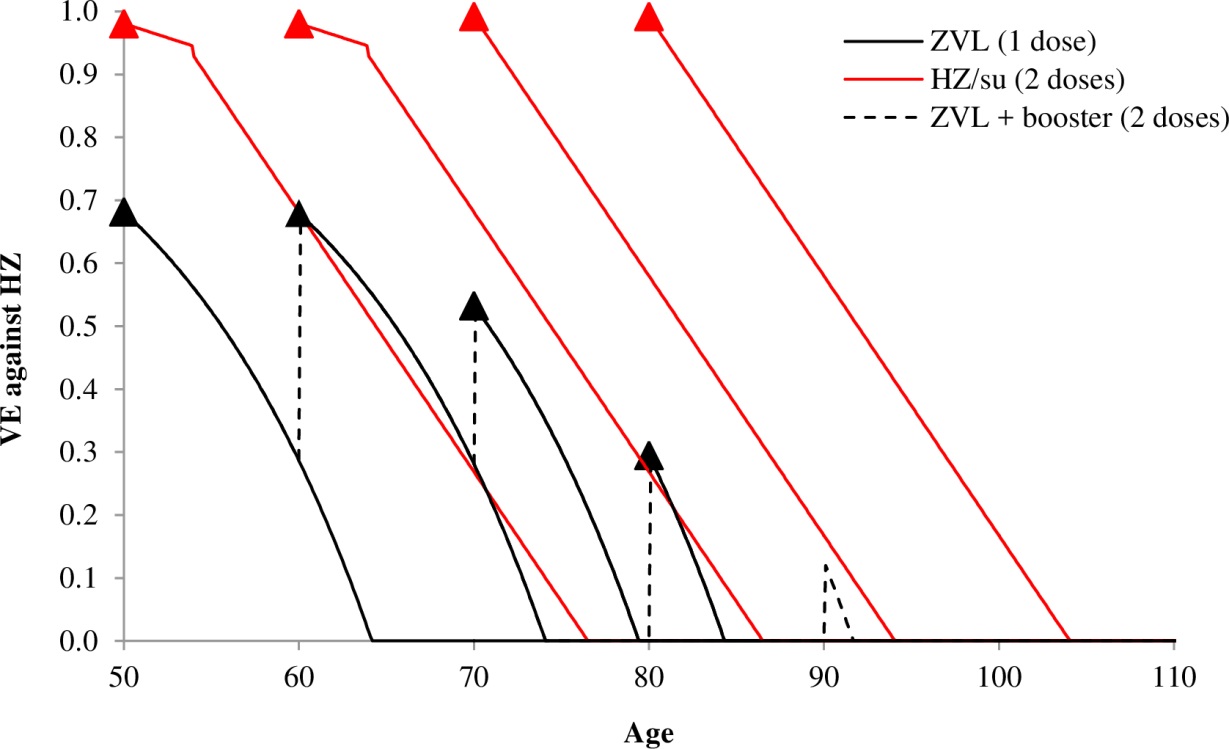


Figure S8: Modelled vaccine efficacy of the herpes zoster subunit vaccine (two doses within 2 months) and zoster vaccine live (single dose, or single dose with booster after 10 years) over time by vaccination age. HZ: Herpes zoster, HZ/su: Herpes zoster subunit vaccine, VE: Vaccine efficacy, ZVL: Zoster vaccine live.

## Sensitivity analysis

We performed several one-way deterministic sensitivity analyses. As the Dutch cost-effectiveness guideline recommends that the lifetime horizon should be long enough to capture all the differential effects of the options [10], we also showed results over a lifetime time-horizon. Additionally, we performed a scenario analysis in which we used incidence estimates of an immunocompetent target-group. Based on published data from Germany and UK, we estimated that the incidence of HZ-related GP visit, hospitalizations and deaths was 0.78, 0.88 and 0.87 times lower among the immunocompetent population as compared with the total population [21, 22]. Subsequently, the Dutch incidence rates were multiplied by these risk ratios. Concerning health effects we explored disutilities of HZ by pain state and total QALY losses per HZ case that have been previously estimated by Van Hoek et al. et al.[23] Moreover, a scenario was performed in which a QALY loss due to grade 3 adverse events at injection-site was included. In the trials, grade 3 adverse events were defined as events that prevented normal everyday activities during 1-3 days. The risk difference of patients with grade 3 adverse events between the vaccine arm and placebo arm was 13.4% for HZ/su and 0.9% for ZVL [14, 15, 17, 24]. The disutility of a grade 3 adverse event was assumed at 0.462 (a shift from no problem with usual activities to severe problems with usual activities on the EQ-5D-3L instrument [3]) and the median duration at 2 days [14]. We assumed that this disutility and duration was also valid for grade 3 adverse events of ZVL. Finally, we varied the discount rates to an internationally accepted 3% for costs and QALYs and to 1.5% for costs and QALYs, according to new guidelines for the evaluation of vaccines by the UK Joint Committee on Vaccination and Immunisation [25].

For HZ/su we performed a scenario analysis in which the adherence of the second dose was altered. Over a limited follow-up period of less than 3 months, the efficacy of a single dose of HZ/su was estimated at 90.1% (95% CI: 58.9-98.8%) for ≥50-year-olds and 69.5% (95% CI: 24.9-89.1%) for ≥70-year-olds, but no data on the waning of efficacy over time was available. Immunogenicity data, however, indicates that one dose of HZ/su induces a considerably lower and less long persisting immune response as compared to two doses [26]. Given the trend that the efficacy of one dose of HZ/su decreases by increasing age and that the waning is expected to be higher as compared to two doses, we assumed that the efficacy of one dose of HZ/su is equal to one dose of ZVL.

For ZVL we conducted multiple scenario analysis that included the use of additional efficacy against PHN among vaccinated HZ cases and effectiveness data from post-licensure studies. The additional efficacy against PHN was obtained from the SPS and was estimated for the age-groups 60-69, 70-79 and ≥80 years separately, using the equation: (VE_PHN_ – VE_HZ_) / (1-VE_HZ_), in which VE is vaccine efficacy [27]. This resulted in an additional efficacy of 4.8% for 60- to 69-year-olds, 55.4% for 70- to 79-year-olds and 25.9% for ≥80-year-olds [17]. These additional efficacies were included in the model by reducing the risk of clinical relevant pain after 3 months of disease onset. We assumed that the waning rate of the additional efficacy against PHN was equal to the waning rate of efficacy against HZ. For the use of ZVL post-licensure effectiveness data, we explored data in our model from the US Kaiser Permanente Northern California over the period 2007-2015, the US Medicare database over the period 2007-2014 and the UK Clinical Practice Research Datalink database over the period 2013-2016 [28-30]. All three studies are population based retrospective cohort studies and represent a study with relatively similar (49% among ≥60-year-olds), lower (33% among ≥65-year-olds) and higher (62% among 68-70-year-olds and 76-79-year-olds) vaccine effectiveness against HZ as compared with the trial. We estimated linear functions on the annual data of the total cohort, which are presented in Figure S9, Figure S10 and Figure S11. Similarly to the efficacy estimates from the trial, we estimated risk ratios by age-group to take into account the difference of vaccine effectiveness at take with age (Table S6).


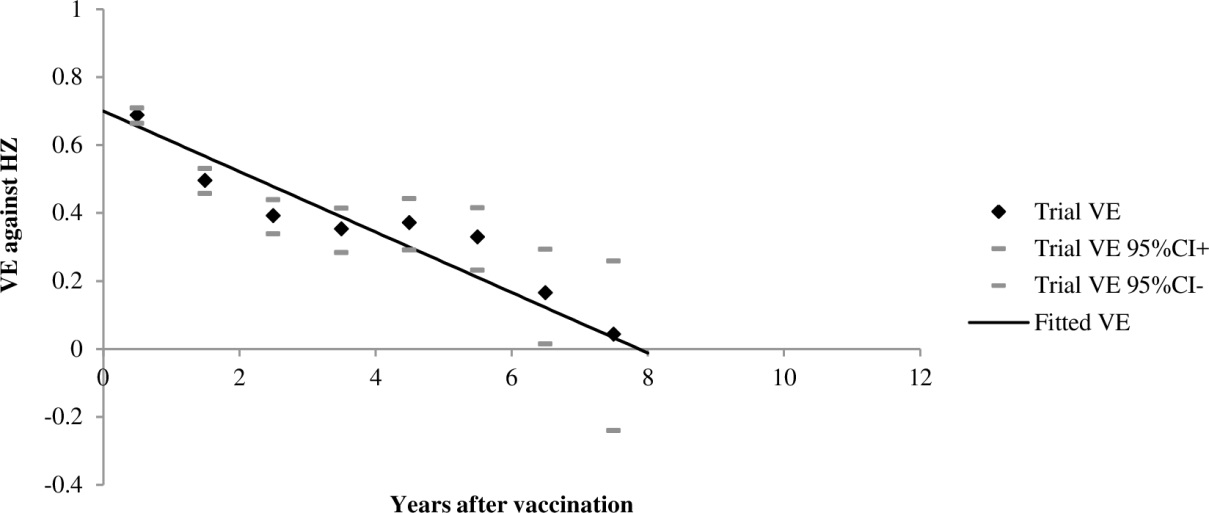


Figure S9: The linear function fitted for the zoster vaccine live using data from the Kaiser Permanente Northern California up to 7 years [28]. The function was estimated at 0.699 (±0.033) – 0.089 (±0.015)*years, with R^2^ = 0.856. HZ: Herpes zoster, VE: Vaccine effectiveness


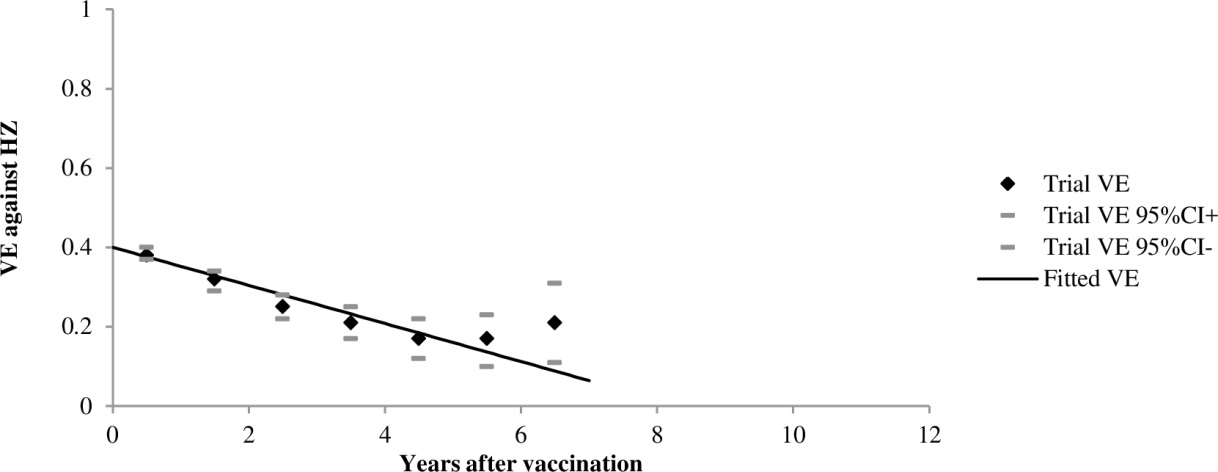


Figure S10: The linear function fitted for the zoster vaccine live using data from the US Medicare database up to 7 years [29]. The function was estimated at 0.397 (±0.013) – 0.048 (±0.006)*years, with R^2^ = 0.928. HZ: Herpes zoster, VE: Vaccine effectiveness


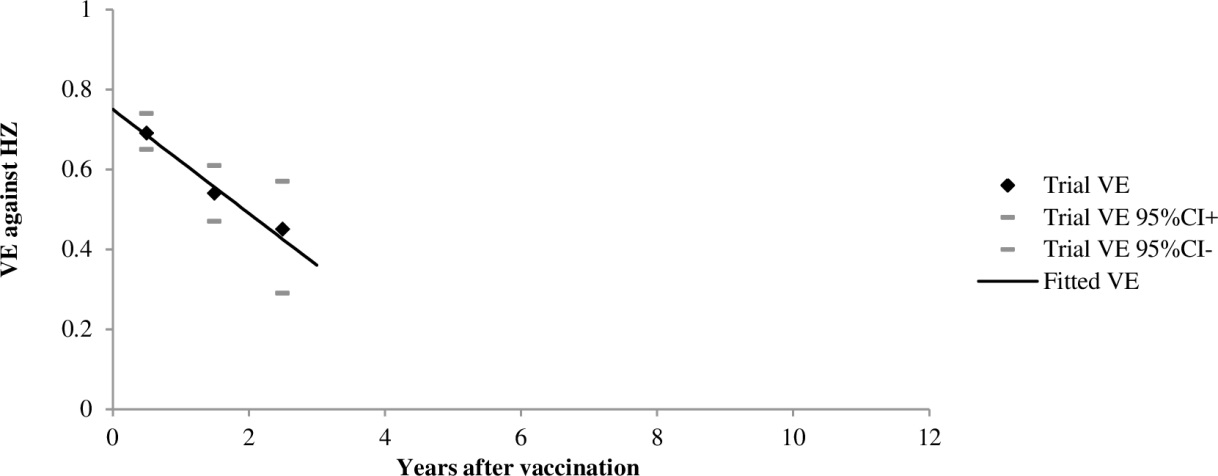


Figure S11: The linear function fitted for the Zoster vaccine live using data from the UK Clinical Practice Research Datalink database up to 3 years [30]. The function was estimated at 0.753 (±0.019) – 0.133 (±0.017)*years, with R^2^ = 0.983. HZ: Herpes zoster, VE: Vaccine effectiveness

Table S6: Fitted risk ratios per age-group of the vaccine effectiveness of the live-attenuated vaccine (Zoster vaccine live [ZVL]) using data from US Kaiser Permanente Northern California, US Medicare database and UK Clinical Practice Research Datalink database [28-30]. These risk ratios were used to adjust the intercept of the fitted function.

| **Variable** | **Tseng, 2016 [28]; US** | **Izurieta, 2017 [29]; US** | **Walker, 2018 [30]; UK** |
| --- | --- | --- | --- |
| Risk ratio of VE by age |  |  |  |
| 60-64 years | 1.143 | NA | NA |
| 65-69 years | 0.980 | 1.091 | NA |
| 70-74 years | 0.959 | 1.061 | 1.00 |
| 75-79 years | 0.959 | 0.970 | NA |
| 80-84 years | 0.857 | 0.939 | 1.03 |
| 85-89 years | 0.857 | 0.970 | NA |
| ≥90 years | 0.857 | 0.970 | NA |

NA: Not available, UK: United Kingdom, US: United States, VE: Vaccine effectiveness.

## References

1. van Wijck AJM, Aerssens YR. Pain, Itch, Quality of Life, and Costs after Herpes Zoster. Pain Pract 2017, 17(6):738-746.

2. Statistics Netherlands. Life-expectancy; Gender, age (per year and period of five years) 2017 http://statline.cbs.nl/Statweb/publication/?DM=SLNL&PA=37360ned&D1=0&D2=0&D3=a&D4=94-95%2cl&HDR=G1%2cT&STB=G2%2cG3&VW=T. Accessed at 1 December 2017.

3. Szende A, Janssen B, Cabases J. Self-Reported Population Health: An International Perspective based on EQ-5D: Springer Open; 2014.

4. Dutch Hospital Data (DHD). Landelijke basisregistratie ziekenhuiszorg (LBZ) [Dutch national registration hospital care] 2017 https://www.dhd.nl/producten-diensten/LBZ/Paginas/Dataverzameling-LBZ.aspx. Accessed at 1 December 2017.

5. Hakkaart-van Roijen L, Essink-Bot ML. Handleiding Vragenlijst over Ziekte en Werk. Rotterdam: Institute for Medical Technology Assessment; 1999.

6. Statistics Netherlands. Absenteeism according to employees; gender and age 2017 http://statline.cbs.nl/Statweb/publication/?DM=SLNL&PA=83056ned&D1=a&D2=0&D3=5-6&D4=l&HDR=G3,T&STB=G1,G2&VW=T. Accessed at 1 December 2017.

7. Statistics Netherlands. Labour participation; Key figures 2016 http://statline.cbs.nl/Statweb/publication/?DM=SLNL&PA=82309NED&D1=22-23&D2=a&D3=18-22&D4=0&D5=69&HDR=G1,T&STB=G2,G3,G4&VW=T. Accessed at 1 December 2017.

8. Eysink PED, Hamberg-van Reenen HH, van Gool CH, Hoeymans N, Burdorf A. Measuring lost years of work due to illness: Disease-Adjusted Working Years (DAWY). Bilthoven: National Institute for Public Health and the Environment; 2010.

9. Koopmanschap MA, Rutten FF, van Ineveld BM, van Roijen L. The friction cost method for measuring indirect costs of disease. J Health Econ 1995, 14(2):171-189.

10. National Health Care Institute. Guideline for economic evaluations in healthcare 2016 https://english.zorginstituutnederland.nl/publications/reports/2016/06/16/guideline-for-economic-evaluations-in-healthcare. Accessed at 1 December 2017.

11. National Health Care Institute. Farmacotherapeutisch Kompas 2017 https://www.farmacotherapeutischkompas.nl/. Accessed at 1 December 2017.

12. van Baal PH, Wong A, Slobbe LC, Polder JJ, Brouwer WB, de Wit GA. Standardizing the inclusion of indirect medical costs in economic evaluations. Pharmacoeconomics 2011, 29(3):175-187.

13. National Health Care Institute. Medicijnkosten.nl 2017 www.medicijnkosten.nl. Accessed at 1 December 2017.

14. Lal H, Cunningham AL, Godeaux O, Chlibek R, Diez-Domingo J, Hwang SJ, Levin MJ, McElhaney JE, Poder A, Puig-Barbera J *et al*. Efficacy of an adjuvanted herpes zoster subunit vaccine in older adults. N Engl J Med 2015, 372(22):2087-2096.

15. Cunningham AL, Lal H, Kovac M, Chlibek R, Hwang SJ, Diez-Domingo J, Godeaux O, Levin MJ, McElhaney JE, Puig-Barbera J *et al*. Efficacy of the Herpes Zoster Subunit Vaccine in Adults 70 Years of Age or Older. N Engl J Med 2016, 375(11):1019-1032.

16. Curran D, Van Oorschot D, Varghese L, Oostvogels L, Mrkvan T, Colindres R, von Krempelhuber A, Anastassopoulou A. Assessment of the potential public health impact of Herpes Zoster vaccination in Germany. Hum Vaccin Immunother 2017, 13(10):2213-2221.

17. Oxman MN, Levin MJ, Johnson GR, Schmader KE, Straus SE, Gelb LD, Arbeit RD, Simberkoff MS, Gershon AA, Davis LE *et al*. A vaccine to prevent herpes zoster and postherpetic neuralgia in older adults. N Engl J Med 2005, 352(22):2271-2284.

18. Schmader KE, Oxman MN, Levin MJ, Johnson G, Zhang JH, Betts R, Morrison VA, Gelb L, Guatelli JC, Harbecke R *et al*. Persistence of the efficacy of zoster vaccine in the shingles prevention study and the short-term persistence substudy. Clin Infect Dis 2012, 55(10):1320-1328.

19. Morrison VA, Johnson GR, Schmader KE, Levin MJ, Zhang JH, Looney DJ, Betts R, Gelb L, Guatelli JC, Harbecke R *et al*. Long-term persistence of zoster vaccine efficacy. Clin Infect Dis 2015, 60(6):900-909.

20. Schmader KE, Levin MJ, Gnann JW, Jr., McNeil SA, Vesikari T, Betts RF, Keay S, Stek JE, Bundick ND, Su SC *et al*. Efficacy, safety, and tolerability of herpes zoster vaccine in persons aged 50-59 years. Clin Infect Dis 2012, 54(7):922-928.

21. Schroder C, Enders D, Schink T, Riedel O. Incidence of herpes zoster amongst adults varies by severity of immunosuppression. J Infect 2017, 75(3):207-215.

22. Hobbelen PH, Stowe J, Amirthalingam G, Miller L, van Hoek AJ. The burden of hospitalisation for varicella and herpes zoster in England from 2004 to 2013. J Infect 2016, 73(3):241-253.

23. van Hoek AJ, Gay N, Melegaro A, Opstelten W, Edmunds WJ. Estimating the cost-effectiveness of vaccination against herpes zoster in England and Wales. Vaccine 2009, 27(9):1454-1467.

24. Dooling KL, Guo A, Patel M, Lee GM, Moore K, Belongia EA, Harpaz R. Recommendations of the Advisory Committee on Immunization Practices for Use of Herpes Zoster Vaccines. MMWR Morb Mortal Wkly Rep 2018, 67(3):103-108.

25. Immunisation and High Consequence Infectious Diseases Team GaPHG. Consultation on the Cost-Effectiveness Methodology for Vaccination Programmes and Procurement (CEMIPP) Report 2018 https://assets.publishing.service.gov.uk/government/uploads/system/uploads/attachment_data/file/683841/Consultation_document_re_CEMIPP.pdf. Accessed at 2018 1 June.

26. Chlibek R, Smetana J, Pauksens K, Rombo L, Van den Hoek JA, Richardus JH, Plassmann G, Schwarz TF, Ledent E, Heineman TC. Safety and immunogenicity of three different formulations of an adjuvanted varicella-zoster virus subunit candidate vaccine in older adults: a phase II, randomized, controlled study. Vaccine 2014, 32(15):1745-1753.

27. Le P, Rothberg MB. Determining the Optimal Vaccination Schedule for Herpes Zoster: a Cost-Effectiveness Analysis. J Gen Intern Med 2017, 32(2):159-167.

28. Tseng HF, Harpaz R, Luo Y, Hales CM, Sy LS, Tartof SY, Bialek S, Hechter RC, Jacobsen SJ. Declining Effectiveness of Herpes Zoster Vaccine in Adults Aged >/=60 Years. J Infect Dis 2016, 213(12):1872-1875.

29. Izurieta HS, Wernecke M, Kelman J, Wong S, Forshee R, Pratt D, Lu Y, Sun Q, Jankosky C, Krause P *et al*. Effectiveness and Duration of Protection Provided by the Live-attenuated Herpes Zoster Vaccine in the Medicare Population Ages 65 Years and Older. Clin Infect Dis 2017, 64(6):785-793.

30. Walker JL, Andrews NJ, Amirthalingam G, Forbes H, Langan SM, Thomas SL. Effectiveness of herpes zoster vaccination in an older United Kingdom population. Vaccine 2018.

# Supplemental results

Cost-effectiveness of vaccination of immunocompetent older adults against herpes zoster in the Netherlands: A comparison between the adjuvanted subunit and live-attenuated vaccine


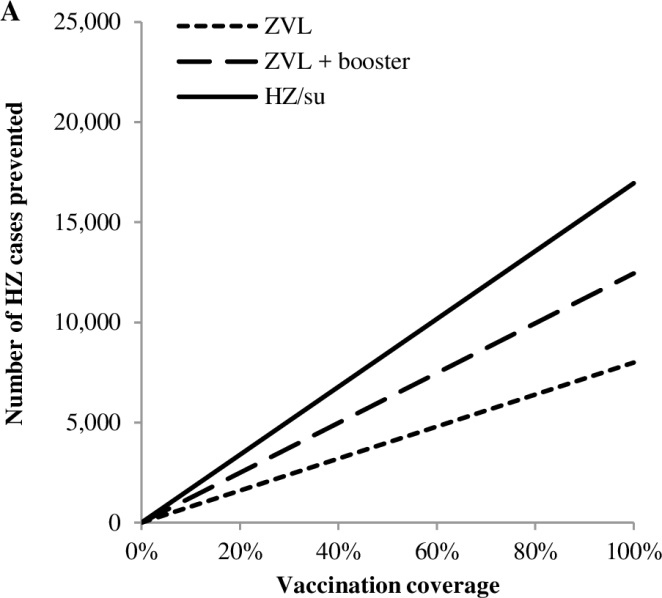

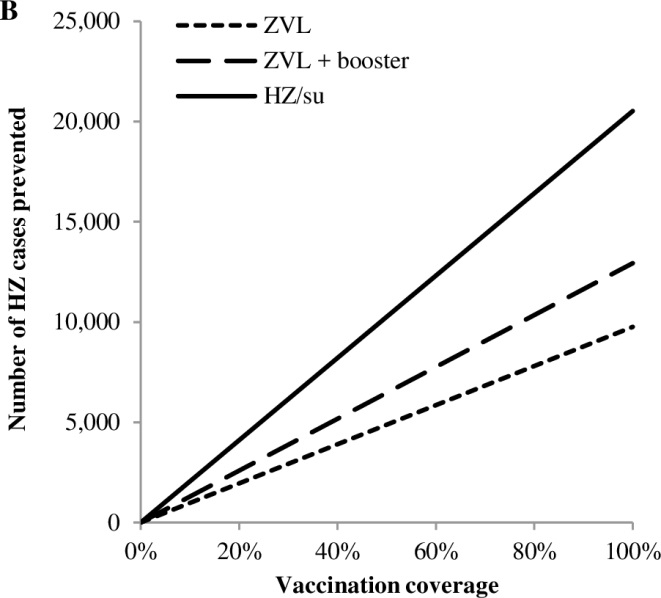


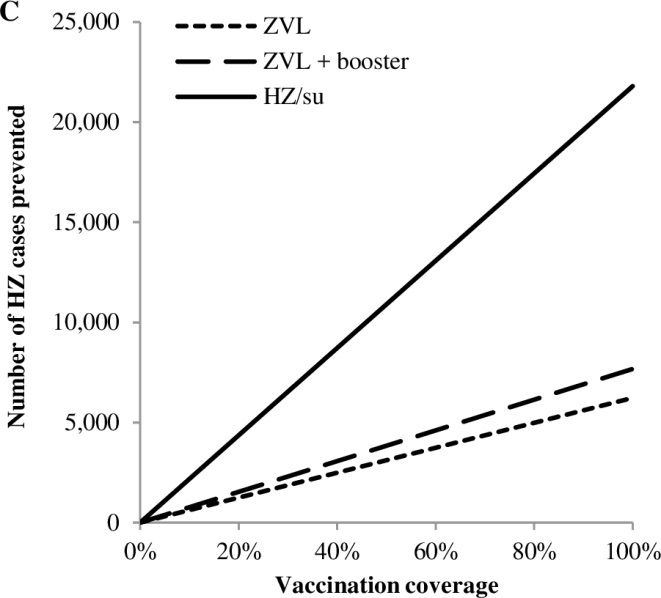

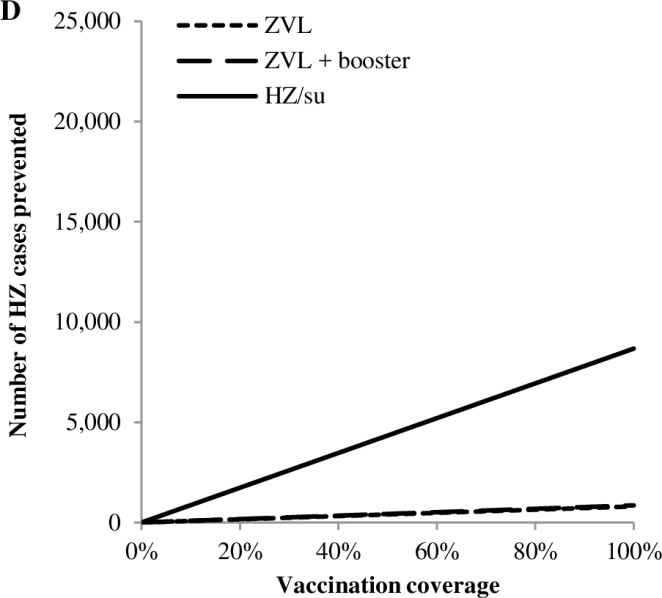


Figure S12: Relation between number of HZ cases prevented and the vaccination coverage for vaccination of A) 50-year-olds, B) 60 year-olds, C) 70 year-olds and D) 80-year-olds over a time-horizon of 15 years. HZ: Herpes zoster, HZ/su: Herpes zoster subunit vaccine, ZVL: Zoster vaccine live.


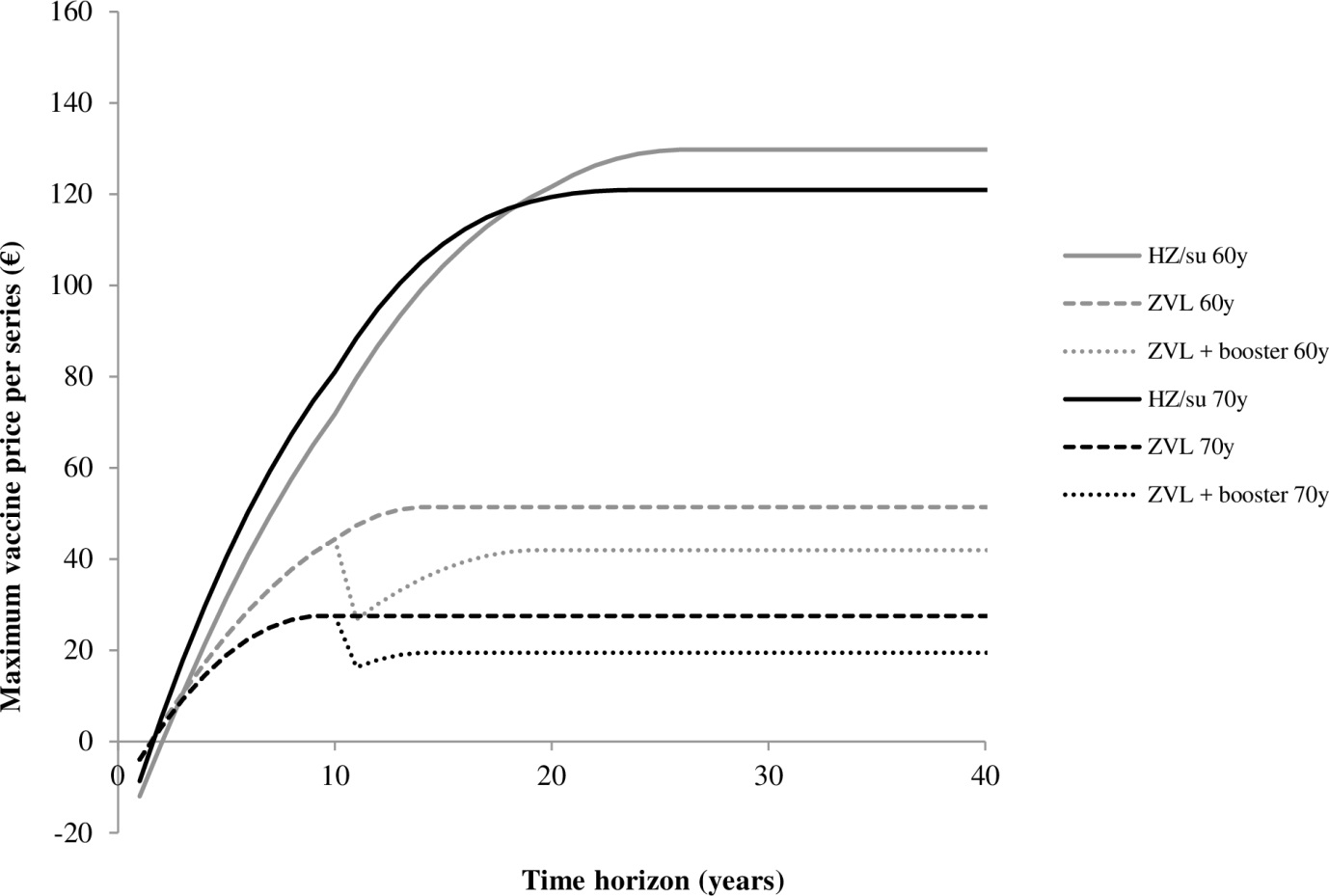


Figure S13: Relation between the time-horizon and the maximum vaccine price to remain below a cost-effectiveness threshold of €20,000 per QALY gained for vaccination of 60-year-olds and 70-year-olds against HZ. Results include a discount rate of 4% for costs and 1.5% for health effects. HZ: Herpes zoster, HZ/su: Herpes zoster subunit vaccine, QALY: Quality-adjusted life year, ZVL: Zoster vaccine live.
