# Supplementary material for: Cost-effectiveness of vaccination of immunocompetent older adults against herpes zoster in the Netherlands: a comparison between the adjuvanted subunit and live-attenuated vaccines
Source: BMC Med. 2018 Dec 6;16:228. doi: 10.1186/s12916-018-1213-5 (PMC6282315; doi:10.1186/s12916-018-1213-5)
Supplement: Supplementary file 3 — AdVISHE checklist. (DOCX 42 kb) [file 12916_2018_1213_MOESM3_ESM.docx]

Cost-effectiveness of vaccination of immunocompetent older adults against herpes zoster in the Netherlands: A comparison between the adjuvanted subunit and live-attenuated vaccine

AdVISHE

Assessment of the ValIdation Status of Health-Economic decision models

AdVISHE is a questionnaire that modellers can complete to report on the efforts performed to improve the validation status of their health-economic (HE) decision model. It is not intended to replace validation by model users but rather to inform the direction of validation efforts and to provide a baseline for replication of the results. In addition to using it after a model is finished, the modellers can use AdVISHE to guide validation efforts during the modelling process.

The modellers are asked to comment on the validation efforts performed while building the underlying HE decision model and afterwards. Many of the questions simply refer to the model documentation. AdVISHE is divided into five parts, each covering an aspect of validation:

- Part A: Validation of the conceptual model (2 questions)
- Part B: Input data validation (2 questions)
- Part C: Validation of the computerized model (4 questions)
- Part D: Operational validation (4 questions)
- Part E: Other validation techniques (1 question)

No final validation score is calculated, as the assessment of the answers and the overall validation effort is left to the model users. It is assumed that the model has been built according to prevailing modelling and reporting guidelines. For instance, the model builders would presumably adhere to the ISPOR-SMDM Modeling Good Research Practices (Caro et al., 2010) and/or the Consolidated Health Economic Evaluation Reporting Standards (CHEERS) Statement (Husereau et al., 2013). Some questions may not be applicable to a particular model. If this is the case, the model builder should take the opt-out option and provide a justification of why this item is not deemed applicable.

Part A: Validation of the conceptual model (2 questions)

Part A discusses techniques for validating the conceptual model. A conceptual model describes the underlying system (e.g., progression of disease) using a mathematical, logical, verbal, or graphical representation. Please indicate where the conceptual model and its underlying assumptions are described and justified.

| The model has been described in the methods section and a graphical representation of the model is shown in the Supplemental Materials Figure 1 |
| --- |

| **A1/ Face validity testing (conceptual model):** Have experts been asked to judge the appropriateness of the conceptual model?  If yes, please provide information on the following aspects:   - Who are these experts? - What is your justification for considering them experts? - To what extent do they agree that the conceptual model is appropriate?   If no, please indicate why not. |
| --- |
| *Yes, the model structure has internally been judged by experts in the field, including health economists, epidemiologists, a virologist and a medical doctor (see list of co-authors). All authors have published multiple papers on the cost-effectiveness of herpes zoster vaccination or on the collection of relevant data for herpes zoster models. The exclusion of rare complications as blindness and deafness was addressed in the limitation section and was judged by a medical expert as very rare among immunocompetent individuals.* |
| Aspects to judge include: appropriateness to represent the underlying clinical process/disease (disease stages, physiological processes, etc.); and appropriateness for economic evaluation (comparators, perspective, costs covered, etc.). |

| **A2/ Cross validity testing (conceptual model):** Has this model been compared to other conceptual models found in the literature or clinical textbooks?  If yes, please indicate where this comparison is reported.  If no, please indicate why not. |
| --- |
| *Yes, the model was developed following a review of health-economic models. A systematic review by the leading author (See De Boer et al. HVI 2014) was updated for this purpose. For the reporting of validation performances we refer to this checklist in the main paper.* |

Part B: Input data validation (2 questions)

Part B discusses techniques to validate the data serving as input in the model. These techniques are applicable to all types of models commonly used in HE modelling.

Please indicate where the description and justification of the following aspects are given:

- search strategy;
- data sources, including descriptive statistics;
- reasons for inclusion of these data sources;
- reasons for exclusion of other available data sources;
- assumptions that have been made to assign values to parameters for which no data was available;
- distributions and parameters to represent uncertainty;
- data adjustments: mathematical transformations (e.g., logarithms, squares); treatment of outliers; treatment of missing data; data synthesis (indirect treatment comparison, network meta-analysis); calibration; etc.

| *We used Pubmed and Google Scholar for the search of relevant data. Each year, an update of the incidence of herpes zoster-related GP visits, hospitalizations and deaths is published in the annual report on infectious diseases by the Dutch National Institute of Public Health and the Environment. The data sources referred to in this report are the best available data sources for the Netherlands. For cost and health-related quality-of-life parameters, we aimed to use Dutch data. A high-quality Dutch prospective study with a long-term follow up of 12 months was used for this purpose, which is known as one of the largest prospective cohort studies with such a long follow-up that is published so far. Assumptions, distributions and data adjustments are described in the main text, input tables and the Supplemental Materials. Main structural assumptions and parameter uncertainty were tested in the sensitivity analyses.* |
| --- |

| **B1/ Face validity testing (input data):** Have experts been asked to judge the appropriateness of the input data?  If yes, please provide information on the following aspects:   - Who are these experts? - What is your justification for considering them experts? - To what extent do they agree that appropriate data has been used?   If no, please indicate why not. |
| --- |
| *Yes, input data have been judged by the co-authors (See A1). Generalizability to the target population was discussed in the discussion, by explaining the potential bias of using data from the total population for an immunocompetent target population. Alternative data sources on this aspect and other input parameters were explored in sensitivity analyses.* |
| Aspects to judge may include but are not limited to: potential for bias; generalizability to the target population; availability of alternative data sources; any adjustments made to the data. |

| **B2/ Model fit testing:** When input parameters are based on regression models, have statistical tests been performed?  If yes, please indicate where the description, the justification and the outcomes of these tests are reported.  If no, please indicate why not. |
| --- |
| *Functions were fitted on the annual vaccine efficacy data points and R^2^ of the fits were reported. Multiple models were used and were visually judged on model fit. For instance, for the adjuvanted subunit vaccine knee- or elbow-bended functions resulted in unrealistic durations of protection.* |
| Examples of regression models include but are not limited to: disease progression based on survival curves; risk profiles using regression analysis on a cohort; local cost estimates based on multi-level models; meta-regression; quality-of-life weights estimated using discrete choice analysis; mapping of disease-specific quality-of-life weights to utility values.  Examples of tests include but are not limited to: comparing model fit parameters (R^2^, AIC, BIC); comparing alternative model specifications (covariates, distributional assumptions); comparing alternative distributions for survival curves (Weibull, lognormal, logit); testing the numerical stability of the outcomes (sufficient number of iterations); testing the convergence of the regression model; visually testing model fit and/or regression residuals. |

Part C: Validation of the computerized model (4 questions)

Part C discusses various techniques for validating the model as it is implemented in a software program. If there are any differences between the conceptual model (Part A) and the final computerized model, please indicate where these differences are reported and justified.

| No differences |
| --- |

| **C1/ External review:** Has the computerized model been examined by modelling experts?  If yes, please provide information on the following aspects:   - Who are these experts? - What is your justification for considering them experts? - Can these experts be qualified as independent? - Please indicate where the results of this review are reported, including a discussion of any unresolved issues.   If no, please indicate why not. |
| --- |
| *The Markov-part of the model has been reviewed by one expert and by one master student, who were independent. These checks were primarily focused on finding bugs.* |
| Aspects to judge may include but are not limited to: absence of apparent bugs; logical code structure optimized for speed and accuracy; appropriate translation of the conceptual model. |

| **C2/ Extreme value testing:** Has the model been run for specific, extreme sets of parameter values in order to detect any coding errors?  If yes, please indicate where these tests and their outcomes are reported.  If no, please indicate why not. |
| --- |
| *Zero testing and high/low input values has been explored for all parameters to test model behaviour.* |
| Examples include but are not limited to: zero and extremely high (background) mortality; extremely beneficial, extremely detrimental, or no treatment effect; zero or extremely high treatment or healthcare costs. |

| **C3/ Testing of traces:** Have patients been tracked through the model to determine whether its logic is correct?  If yes, please indicate where these tests and their outcomes are reported.  If no, please indicate why not. |
| --- |
| *The model was specified to the population level and not to the individual level.* |
| In cohort models, this would involve listing the number of patients in each disease stage at one, several, or all time points (e.g., Markov traces). In individual patient simulation models, this would involve following several patients throughout their natural disease progression. |

| **C4/ Unit testing:** Have individual sub-modules of the computerized model been tested?  If yes, please provide information on the following aspects:   - Was a protocol that describes the tests, criteria, and acceptance norms defined beforehand? - Please indicate where these tests and their outcomes are reported.   If no, please indicate why not. |
| --- |
| *The model was built in Excel. Sub-modules were tested by turning them on and off, comparable to extreme value testing. Macros were walked through step by step to detect any errors in the code.* |
| Examples include but are not limited to: turning sub-modules of the program on and off; altering global parameters; testing messages (e.g., warning against illegal or illogical inputs), drop-down menus, named areas, switches, labelling, formulas and macros; removing redundant elements. |

Part D: Operational validation (4 questions)

Part D discusses techniques used to validate the model outcomes.

| **D1/ Face validity testing (model outcomes):** Have experts been asked to judge the appropriateness of the model outcomes?  If yes, please provide information on the following aspects:   - Who are these experts? - What is your justification for considering them experts? - To what extent did they conclude that the model outcomes are reasonable?   If no, please indicate why not. |
| --- |
| *Experts (see co-author list) have judged the model outcomes on validity.* |
| Outcomes may include but are not limited to: (quality-adjusted) life years; deaths; hospitalizations; total costs. |

| **D2/ Cross validation testing (model outcomes):** Have the model outcomes been compared to the outcomes of other models that address similar problems?  If yes, please provide information on the following aspects:   - Are these comparisons based on published outcomes only, or did you have access to the alternative model? - Can the differences in outcomes between your model and other models be explained? - Please indicate where this comparison is reported, including a discussion of the comparability with your model.   If no, please indicate why not. |
| --- |
| *Yes, in the discussion section a comparison has been made to models addressing the same research question of cost-effectiveness of herpes zoster vaccination that were published in peer-reviewed journals. Explanations of differences were reported. Moreover, a complete list of input differences between two earlier published cost-effectiveness studies of the herpes zoster live-attenuated vaccine in the Netherlands was drafted, but not shown in order to increase the readability of the manuscript.* |
| Other models may include models that describe the same disease, the same intervention, and/or the same population. |

| **D3/ Validation against outcomes using alternative input data:** Have the model outcomes been compared to the outcomes obtained when using alternative input data?  If yes, please indicate where these tests and their outcomes are reported.  If no, please indicate why not. |
| --- |
| *A scenario analysis was performed in which data from several other sources were explored in the model and the outcomes were reported in a tornado diagram. Other sources included for instance the use of effectiveness data.* |
| Alternative input data can be obtained by using different literature sources or datasets, but can also be constructed by splitting the original data set in two parts, and using one part to calculate the model outcomes and the other part to validate against. |

| **D4/ Validation against empirical data:** Have the model outcomes been compared to empirical data?  If yes, please provide information on the following aspects:   - Are these comparisons based on summary statistics, or patient-level datasets? - Have you been able to explain any difference between the model outcomes and empirical data? - Please indicate where this comparison is reported.   If no, please indicate why not.  **D4.A/** Comparison against the data sources on which the model is based (dependent validation). |
| --- |
| *Yes, the number of clinical events (cases, hospitalizations, deaths) were compared to the incidence rates of the original sources on which the analysis was based (summary statistics). Differences in absolute number of cases could be explained by taking into account false positive cases or misclassification of herpes zoster-related mortality.* |
| **D4.B/** Comparison against a data source that was not used to build the model (independent validation). |
| *No comparison to external data sources was done as no other Dutch data sources were identified, but incidence of clinical events was found to be similar among European countries, implying that our analysis might provide useful information for decision makers of other countries as well.* |

Part E: Other validation techniques (1 question)

| **E1/ Other validation techniques:** Have any other validation techniques been performed?  If yes, indicate where the application and outcomes are reported, or else provide a short summary here. |
| --- |
| *Structured walk-throughs were performed for the Markov part of the model and naïve benchmarking was performed* |
| Examples of other validation techniques: structured “walk-throughs” (guiding others through the conceptual model or computerized program step-by-step); naïve benchmarking (“back-of-the-envelope” calculations); heterogeneity tests; double programming (two model developers program components independently and/or the model is programmed in two different software packages to determine if the same results are obtained). |
